# *Hematoloechus* sp. attachment shifts endothelium in vivo from pro- to anti-inflammatory profile in *Rana pipiens*: evidence from systemic and capillary physiology

**DOI:** 10.1152/ajpregu.00041.2023

**Published:** 2023-06-05

**Authors:** Donna A. Williams, Mary H. Flood

**Affiliations:** College of Nursing, Montana State University, Bozeman, Montana, United States

**Keywords:** hydration, lower vertebrates, nutrition, pulmonary, parasitology

## Abstract

This prospective, descriptive study focused on lung flukes (*Hematoloechus* sp., *H*) and their impact on systemic and individual capillary variables measured in pithed *Rana pipiens*, a long-standing model for studies of capillary physiology. Three groups were identified based on *Hematoloechus* attachment: no *Hematoloechus* (No *H*), *Hematoloechus* not attached (*H* Not Att), and *Hematoloechus* attached (*H* Att). Among 38 descriptive, cardiovascular, and immunological variables, 18 changed significantly with *H*. Symptoms of *H* included weight loss, elevated immune cells, heart rate variability, faster coagulation, lower hematocrit, and fluid accumulation. Important capillary function discoveries included median baselines for hydraulic conductivity (*L*_p_) of 7.0 (No *H*), 12.4 (*H* Not Att), and 4.2 (*H* Att) × 10^−7^ cm·s^−1^·cmH_2_O^−1^ (*P* < 0.0001) plus seasonal adaptation of sigma delta pi [σ(π_c_–π_i_), *P* = 0.03]. Pro- and anti-inflammatory phases were revealed for *L*_p_ and plasma nitrite/nitrate concentration ([NO_x_]) in both *H* Not Att and *H* Att, whereas capillary wall tensile strength increased in the *H* Att. *H* attachment was advantageous for the host due to lower edema and for the parasite via a sustained food source illustrating an excellent example of natural symbiosis. However, *H* attachment also resulted in host weight loss: in time, a conundrum for the highly dependent parasite. The study increases overall knowledge of *Rana pipiens* by revealing intriguing effects of *H* and previously unknown, naturally occurring seasonal changes in many variables. The data improve *Rana pipiens* as a general scientific and capillary physiology model. Diseases of inflammation and stroke are among the clinical applications.

## INTRODUCTION

For over a century, *Rana pipiens* (North American leopard frog) has been a reliable animal model used for a wide variety of scientific investigations into organ and tissue physiology as well as for student laboratory demonstrations. In the United States, *Rana pipiens* are caught in the wild and sold by companies located primarily in the eastern half of the country. In our laboratory, *Rana pipiens* was selected to study capillary physiology as it is a lower vertebrate that spawned and matured in a natural environment.

The lung fluke *Hematoloechus* sp. (*H*) was reported first in the early 19th century. *H* have a complicated taxonomy and at least 89 species of the genus are known to exist (1). In general, *H* have a life cycle with two intermediate hosts, and anurans are its definitive host (2). *H* eggs are consumed by the first intermediate host, the air-breathing freshwater snail (planorbid). Within the snail, the eggs hatch into sporocysts that produce cercaria, the larval form of trematodes. Cercaria are then shed from the snail and enter dragonflies or damselflies (odonata). When a frog (or toad) eats a dragonfly or damselfly and if that odonate has cercaria, the parasite makes its way through the esophagus and lodges in the lungs within 5 days. Once the lung fluke is located in its definitive host, the life cycle of *H* is complete (3). In its natural habitat, *Rana pipiens* is an opportunistic feeder, eating a variety of invertebrates including ants, beetles, and dragonflies. *Rana pipiens* is a definitive host of *H* (4). Although the parasite itself is well studied, very little is known about how *H* effect *Rana pipiens*.

For studies of capillary function (hydraulic conductivity, water permeability, *L*_p_), *Rana pipiens* has long been considered a gold standard model. Data from frog capillaries have been published for decades in primary and review articles (5–7). Frog capillary data have been used to test mathematical models of permeability (8) and have provided the target baseline for *L*_p_ in cultured endothelial monolayers (9). This long history and broad application obligates investigations into new aspects of the model to better understand results from past, present, and future studies.

The mesentery is a thin, transparent tissue juxta-intestines that can be exteriorized with minimal trauma in *Rana pipiens* allowing access to its network of capillaries. Location of each capillary within the microvascular network is known making the model a useful bridge between cultured endothelial cells and whole organ studies of permeability. Aided by an inverted light microscope, individual capillaries are cannulated in situ with a glass micropipette, and the volume flux (*J*_v_) and surface area (*S*) of each capillary are measured directly. Blood perfusion of each capillary is maintained up to the moment of cannulation, and neural input and lymph flow remain intact. The approach was first used by Landis in 1927 (10) and later modified by Michel and colleagues (11). In the past, *L*_p_ values <10.0 × 10^−7^ cm·s^−1^·cmH_2_O^−1^ have been used by some investigators as criteria for including capillary *L*_p_ results in the literature (12). Capillaries with *L*_p_ values >10.0 × 10^−7^ cm·s^−1^·cmH_2_O^−1^ have been considered “inflamed.” This exclusion criteria for single capillary data has not been substantiated by objective physiological indicators of inflammation.

The purpose of the present study was to evaluate the impact of lung *H* on systemic and capillary variables measured in *Rana pipiens*. Baseline time series data were collected across the year for each variable to establish a useful reference. A secondary analysis was performed on statistical outliers of capillary *L*_p_ to identify underlying physiological correlates with high *L*_p_ values.

## MATERIALS AND METHODS

### Animal Procedures

Wild-caught *Rana pipiens* [male; *n* = 422 (No *H* = 228; *H* Not Att = 100; *H* Att = 94); weight = 34.8 (SD 6.0) g; length = 19.7 (SD 0.8) cm; # *H* (median (± 25%/75%) = 2 (± 1/4), range = 1–22); 22 samples over 7 yr; Charles D. Sullivan Co., Nashville, TN] were determined to be healthy by visual inspection. Upon arriving at the animal care facility, each frog was examined and placed in a gentamicin (1.3 mg·mL^−1^) bath for the first 24 h. The frogs were then moved to containers with dry areas and fresh, dechlorinated water that was filtered continuously. The frogs were cage sedentary and housed six per container. Containers were placed on racks in a room where the environment was maintained at 15°C, and light was controlled on a 12-h:12-h light:dark cycle. The frogs were fed weekly. Once every 2 weeks they were fed individually using a 1-mL syringe filled with vegetable beef baby food (Gerber), and on the alternate week, crickets were released into each container. Data were collected from 2010 to 2017. Protocols were approved and monitored by the Animal Care and Use Committee at Montana State University, Bozeman, MT.

### Surgery and Mesenteric Tissue Preparation

Each frog was pithed cerebrally, and one drop of blood was placed on a glass slide for measurement of clotting time. Cotton was placed in the cranial cavity to separate cerebral and spinal column nerve tissue. Body weight, length, and displaced volume were measured, and the frog was placed supine on a pad and heart rate was counted. An incision was made through the skin and muscle wall to expose the contents of the abdomen. A loop of small intestine was exteriorized and draped carefully over a quartz pillar, which allowed the mesenteric tissue to lay flat atop the pillar. The entire tissue preparation was kept cool (14°C–16°C, type-T thermocouple wire, Digi-Sense, Cole Parmer, Vernon Hills, IL) and moist with frog Ringer solution superfused continuously (≈ 3 mL·min^−1^) from a reservoir and siphoned away with a vacuum line. The tissue and its microvascular bed remained intact, attached to the frog, and blood-perfused during the entire protocol.

### Blood and Lymph Fluid Collection

Just before the mesenteric tissue was exposed, a small incision was made in the skin to collect lymph fluid for later analysis of skin lymph protein concentration ([skpr]). After the area between the skin and abdominal wall was carefully swabbed dry, blood was drawn into three heparinized microhematocrit tubes (74 µL each; not less than 2 USP units of ammonium heparinate per tube; Thermo Fisher Scientific) from a peripheral vein located along the interior surface of the skin near the right axilla and exterior to the abdominal cavity. The first sample was used for measurement of systemic hematocrit (sysHct) and plasma protein concentration ([plpr]), the second for systemic red blood cell concentration ([RBC]), white blood concentration ([WBC]), activated white blood concentration (a[WBC]), and hemoglobin concentration ([Hb]), and the third was prepared for measurement of plasma nitrite/nitrate concentration ([NO_x_]). Next, a small incision was made in the abdominal wall, and lymph fluid was collected for later analysis of abdominal cavity fluid protein concentration ([abpr]). Surgical and body fluid sampling procedures have been published previously (13–17).

### Necropsy

After the experimental protocol was completed, each animal was euthanized and a detailed necropsy was performed. Lungs and internal organs were inspected for parasites and signs of distress. If present, *H* in the lungs were removed and counted (18). Lungs, spleen, and liver were dissected free, blotted dry, and weighed.

### Solutions

Frog Ringer solution was prepared fresh daily from 5× concentrated stock (pH 7.4 at 15°C) to the following concentrations (mM): 111.0 NaCl, 2.4 KCl, 1.0 MgSO_4_, 1.1 CaCl_2_, 5.0 glucose, 2.0 NaHCO_3_, and 5.0 HEPES/Na-HEPES. For the pipette solution, bovine serum albumin (BSA) was dialyzed (6,000– 8,000 MWCO, Spectra/Por membrane, Spectrum, Houston, TX) and dissolved in frog Ringer (10 mg·mL^−1^; A-4378, Sigma Chemical Co., St. Louis, MO). BSA was made fresh daily, mixed with human red blood cells (hRBCs; 1–3% hematocrit prepared as described previously, 15), and kept on ice until use. The same BSA lot number and human donor were used for all experiments to minimize variability.

### Measures of Systemic Variables in Blood and Lymph Fluid

#### Clotting time.

Coagulation of a single drop of blood was determined visually. *Time 0* for the clotting time measurement was designated as the time when the blood contacted the glass slide. The drop was observed continuously until a clot formed. Time to clot formation was recorded.

#### Systemic hematocrit.

Hematocrit tubes were double sealed with critoseal (Thermo Fisher Scientific) and spun within minutes of blood collection at 13,460 *g* for 3 min (IEC Micro-MB Microcentrifuge fitted with microhematocrit rotor). sysHct was measured using a microcapillary reader (IEC, Needham Heights, MA) placed consistently at the same height and angle for each measurement with the indicator line placed at the interface of the red blood cell (RBC) column and white blood cell (WBC) layer. Intrasample reliability was within 1%.

#### Red blood cell concentration.

A 5-µL sample of whole blood was diluted 1:200 in filtered (0.45 µm pore size, Millipore, Billerica, MA) frog Ringer solution. Diluted blood (10 µL) was loaded into a Reichert bright line hemacytometer (Improved Neubauer ruling pattern, Cat. No. 1483, Hausser Scientific, Horsham, PA). Red blood cells (RBCs) were counted (Fisher Laboratory counter) using a Zeiss light microscope (Axiostar Plus, ×10 A-plan objective). RBCs located completely within the ruling lines of the hemacytometer were included in the count. If a portion of a cell was located outside the line it was not counted. Mean corpuscular volume (MCV) was calculated as MCV = (sysHct/[RBC]) × 10^7^.

#### White blood cell concentration.

The [RBC] procedure was followed for [WBC] with the only change being a 1:20 dilution of a 12.5 µL whole blood sample. The diluted sample was loaded onto the Neubauer slide, and WBCs were differentiated by size (basophils, small; neutrophils, medium; lymphocytes, large) and counted. To measure concentration of activated white blood cells ([aWBC]), a solution of nitroblue tetrazolium dye was added to 12.5 µL of whole blood and incubated at 37°C for 20 min and then at room temperature for 20 min. The dye/whole blood sample was then diluted 1:20 with frog Ringer, loaded onto the Neubauer slide, and inspected for [aWBC], defined as those that had burst after taking up the dye (19). The same counting decision rules were used as for [RBC].

#### Hemoglobin concentration.

[Hb] was determined from a 20-µL sample of whole blood mixed with 480 µL of Drabkin’s Reagent (Sigma Chemical Co.) in a glass test tube. A standard curve was prepared with each assay using cyanomethemoglobin (range 20–80 mg·dL^−1^, Stanbio Diagnostics Co., Boerne, TX). One milliliter of the blood/Drabkin’s mixture or standards solution was pipetted into microcuvettes and read on a spectrophotometer (Turner SP-850, Barnstead/Thermolyne, Dubuque, IA) at a wavelength of 540 nm. Absorbance was recorded, and [Hb] calculated from the standard curve. Mean corpuscular hemoglobin concentration ([MCHb]) calculation was [MCHb] = ([Hb]/sysHct) × 100.

#### Plasma [NOx].

Samples of whole blood were placed immediately into EDTA tubes, put on wet ice, and centrifuged (Hettich MIKRO 22 R, Proscientific, Oxford, CT) within 1 h at 3,200 *g* for 15 min (4– 5°C). Plasma was removed, injected into siliconized vacuum tubes, and stored at −70°C.

Within 2 to 3 mo of sample collection, [NO_x_] was measured using chemiluminescence (Sievers Nitric Oxide Analyzer; model 280i, Boulder, CO; sensitivity, 1 pmole·mL^−1^) as reported previously (20). Briefly, each plasma sample (5–200 µL) was heated to 96°C and injected into a receptacle, which contained a freshly made solution of 0.1 M vanadium III chloride (5 mL, Aldrich Chemical Co., Milwaukee, WI) in 3.0 M HCl (Ricca Chemical Co., Arlington, TX). Vanadium III reduces nitrite to nitric oxide at room temperature (20°C) and reduces NO_x_ to nitric oxide at 85°C (21, 22). Area under the curve generated by the nitric oxide analyzer was integrated and recorded on a Dell computer (Pentium 4, GX260 Optiplex). The nitric oxide analyzer was calibrated with KNO_3_ standards (range 0.5–5.0 nM). Water was collected from each housing container, and [NO_x_] measured to assure consistent housing conditions.

#### Protein concentrations [plpr], [skpr], and [abpr].

The plasma portion of the first hematocrit tube sample (see *Blood and Lymph Fluid Collection*) and the lymph fluid samples were stored at −20°C for no more than 2 mo. A microassay (BioRad Laboratories, Inc., Hercules, CA) was used to measure protein concentration according to the manufacturer’s instructions (23). A standard curve and duplicate samples for each animal were read at 595 nm bandwidth (Turner SP-850 spectrophotometer, Barnstead/Thermolyne, Dubuque, IA).

### Measures of Capillary Variables in Mesenteric Tissue

#### Intravital video microscopy and video imaging.

Each mesentery was transilluminated to visualize capillaries using an inverted, compound microscope (UM ×10 long working distance objective, 0.22 NA, Diavert, Leitz). Real-time video recordings were captured for each experiment (Panasonic AG-6300, Matsushita Electric Industries, JN) with time (0.01 s, VTG-33, For-A, JN) superimposed onto the image (final magnification, ×500). Video clips were digitized (media converter DVMC-DA2, Sony) and archived (LaCie, Ltd). Measurements were calibrated daily to a stage micrometer (0.01 mm, Meiji Techno, JN). The microscope assembly and glass microtools used for cannulation of individual capillaries have been described previously (13–17).

#### Capillary identification.

Individual mesenteric capillaries were defined as tubes of endothelial cells situated between branches, free of vascular smooth muscle, and devoid of rolling or sticking WBCs. The true capillaries (TCs) used in this study were identified in situ by the direction of blood flow, which diverged upstream and converged downstream at the ends of each capillary tube (24).

#### Capillary tube hematocrit (tHct).

Frog red blood cell (fRBC) count per 500-µm length of the capillary and capillary radius (*d*/2 = *r*, µm) were used to calculate tHct. Capillary diameter (*d*, µm) was obtained from the average of three sites spaced ≈ 50 µm apart on the video recording:

(*1*) $$\text{tHct }\left(\%\right)=\left(\text{fRBC}\times \text{294}.\text{4}\times \text{1}00\right)/\left(\text{3}.\text{14}\times \left(r^{\text{2}}\right)\times \text{5}00\right)$$

#### Capillary red blood cell flux.

To measure RBC flux, a point along the capillary segment was selected on the video recording. The recording was advanced to determine the time (*t*, s) required for 50 fRBC to pass by the designated point. Flux was calculated as:

(*2*) $$\text{RBC Flux }\left({\text{cells}\cdot \text{s}}^{-\text{1}}\right)=\text{5}0/\left(t^{\text{1}}-t^{\text{2}}\right).$$

#### Capillary fluid shear rate and flow.

For each capillary, capillary fluid shear rate (γ) was calculated from fRBC or hRBC velocity (*v*) and *r* (µm). Instantaneous velocity (*v*^i^) of RBCs was measured directly on the video monitor at 15-s intervals, averaged, and calculated as described previously (13–17):

(*3*) $$v^{\text{i}}({\mu \text{m}\cdot \text{s}}^{-\text{1}})=\text{d}x/\text{d}t$$

where *x* was the distance (µm) traveled by a RBC in time (*t*, s). To obtain the mean RBC velocity, *v*^i^ was divided by a correction factor (CF; 11; RBC radius, *R*, µm), assuming that each RBC was centered within the capillary tube.

(*4*) $$\text{CF}=\text{2}\left(\text{1}-\left[{\left(R\right)}^{\text{2}}/\text{2}{\left(r\right)}^{\text{2}}\right]\right)$$

(*5*) $$v({\text{mm}\cdot \text{s}}^{-1})=v^{i}/\text{CF}$$

(*6*) $$\gamma \left({\text{s}}^{-\text{1}}\right)=\text{4}v/r.$$

Capillary blood flow was calculated as:

(*7*) $$\text{Flow }\left({\text{nl}\cdot \text{s}}^{-1}\right)=\left(\text{3}.\text{14}\times v\times d^{\text{2}}\right)/\text{4},000$$

#### Capillary balance pressure.

Immediately following a successful cannulation, the water manometer that was attached to the pipette assembly to maintain pressure and flow was lowered slowly. The pressure at which the flow of hRBC ceased was recorded as capillary balance pressure.

#### Capillary volume flux per surface area.

The modified Landis technique (10, 11) was used to measure volume flux per surface area (*J*_v_/*S*) at two pressures (20 and 30 cmH_2_O) during the first occlusion following a uniform, square wave change in shear stress (Δτ) stimulus (see *Experimental Protocol*). Successful occlusion of each capillary was determined by visual inspection to insure valid measures of *J*_v_. *J*_v_/*S* was calculated from hRBC velocity (d*x*/d*t*^occlusion^, cm·s^−1^), capillary length (*x*_o_, cm), and capillary volume to *S* ratio (*r*/2, cm, assuming cylindrical geometry of each capillary):

(*8*) $$J_{\text{v}}/S\left({\text{cm}\cdot \text{s}}^{-\text{1}}\right)=\left(\text{d}x/\text{d}t^{\text{occlusion}}\right)\left(\text{1}/x_{\text{o}}\right)\left(r/\text{2}\right).$$

To increase precision, d*x*/d*t*^occlusion^ and *x*_o_ were measured on three hRBC (spaced ≈ 50 µm apart) at three time points (2.0, 2.3, and 2.6 s). Three measures of diameter were obtained at three time points to verify that *S* remained constant (13–17).

#### Capillary L_p_ (cm·s^−1^·cmH_2_O^−1^).

The Starling equation assumes a linear relationship between *J*_v_/*S* and capillary pressure (P_c_). Slope of the regression equation for *J*_v_/*S* and P_c_ is *L*_p_:

(*9*) $$J_{\text{v}}/S=L_{\text{p}}[\left({\text{P}}_{\text{c}}-{\text{P}}_{\text{i}}\right)-\sigma (\pi_{\text{c}}-\pi_{\text{i}})]$$

where (P_c_–P_i_) and (π_c_–π_i_) are hydrostatic (P) and oncotic (π) pressure differences between capillary lumen (c) and interstitium (i). Sigma (σ) is the reflection coefficient of the capillary wall to protein [for assumptions see Williams (15)]. σ(π_c_–π_i_) is the *x*-axis intercept of the P_c_–*J*_v_/*S* relationship. Capillary *L*_p_ was calculated for each of the nine measures of *J*_v_/*S* at two pressures (see above) and averaged.

#### Capillary burst pressure.

The first occlusion was maintained after a sufficient recording of the hRBC marker cells for *J*_v_/*S* measurements. Capillary pressure was then uniformly raised by 1 cmH_2_O every 10 s until one or more hRBCs burst through the capillary wall. The capillary was observed closely on the video monitor and through the microscope to ensure accurate assessment. If a hRBC escaped at the occlusion site (an infrequent occurrence), that measurement was excluded.

### Experimental Protocol

Frogs were fasted for 4 days before each experiment. Fresh water was available ad libitum to prevent dehydration. Frogs with one or more [aWBC] were excluded from the study.

After blood collection and surgical procedures, one capillary per frog was cannulated at 10 cmH_2_O (7.4 mmHg), and the balance pressure was measured (see above). The pressure was raised by 1 cmH_2_O to establish low flow during a 2 min equilibration period (*steady state 1*). At the end of equilibration, the capillary was stimulated mechanically with a square wave change in shear stress (Δτ) via an abrupt change in pressure to 30 cmH_2_O (22.1 mmHg). The higher τ was maintained for 2 min (*steady state 2*). *J*_v_/*S* was measured followed by measurement of burst pressure. At the end of the protocol, the animal was euthanized and a necropsy performed.

τ curves were generated for each capillary from video recordings to verify square wave stimulus and plateaus at *steady states 1* and *2*. Δτ for a given capillary was calculated from plateau values as Δτ = τ^steady state 2^–τ^steady state 1^. A physiological range for Δτ occurred in accordance with the downstream resistance of the microcirculation, which varied in situ from animal to animal. In general, it was assumed that filtration during occlusion reflected filtration at *steady state 2*. An important advantage of this technique was that the downstream end of each capillary remained undisturbed, and the Δτ stimulus was applied uniformly to each intact capillary (13–17).

To minimize variability, measurement error, and bias throughout the study, the PI performed all surgical, video microscopy, and capillary protocols, collected body fluid samples, and measured sysHct and heart rate. The research associate measured weights, clotting time, [RBC], [WBC], [Hb], plasma [NO_x_], protein concentrations, capillary diameter, tHct, RBC flux, RBC velocity, and *J*_v_/*S* plus performed necropsies.

### Statistical Analyses

JMP software (SAS Institute, Inc.) was used for statistical analyses. The dataset was control data pooled from experiments performed over the course of 7 years. *n* equaled number of animals. Normality was assessed for each variable using Shapiro–Wilk *W* test, and central tendency reported as means (SD) or median (± 25 and 75% quartiles) as appropriate. Values identified using box and whiskers plots were designated as outliers and excluded. A secondary analysis of *L*_p_ outliers and associated variables was reported. Time series data included two or more data points obtained in 2 or more years. Single time course data points were included in the figures but not in the analyses. One-way analysis of variance with parasite or month as the main effect was used to determine differences between means followed by Tukey’s post hoc tests when the main effect was significant. Differences between medians were determined using van der Waerden test. In the case of unequal variance (Bartlett test), a Welch analysis of variance was employed. Significance was set a priori at *P* ≤ 0.05.

## RESULTS

Averages and time series data are presented below for the three parasite conditions. Results are organized according to systemic and capillary variables.

### Systemic Variables

#### Body and organ weights.

**Figure 1.**
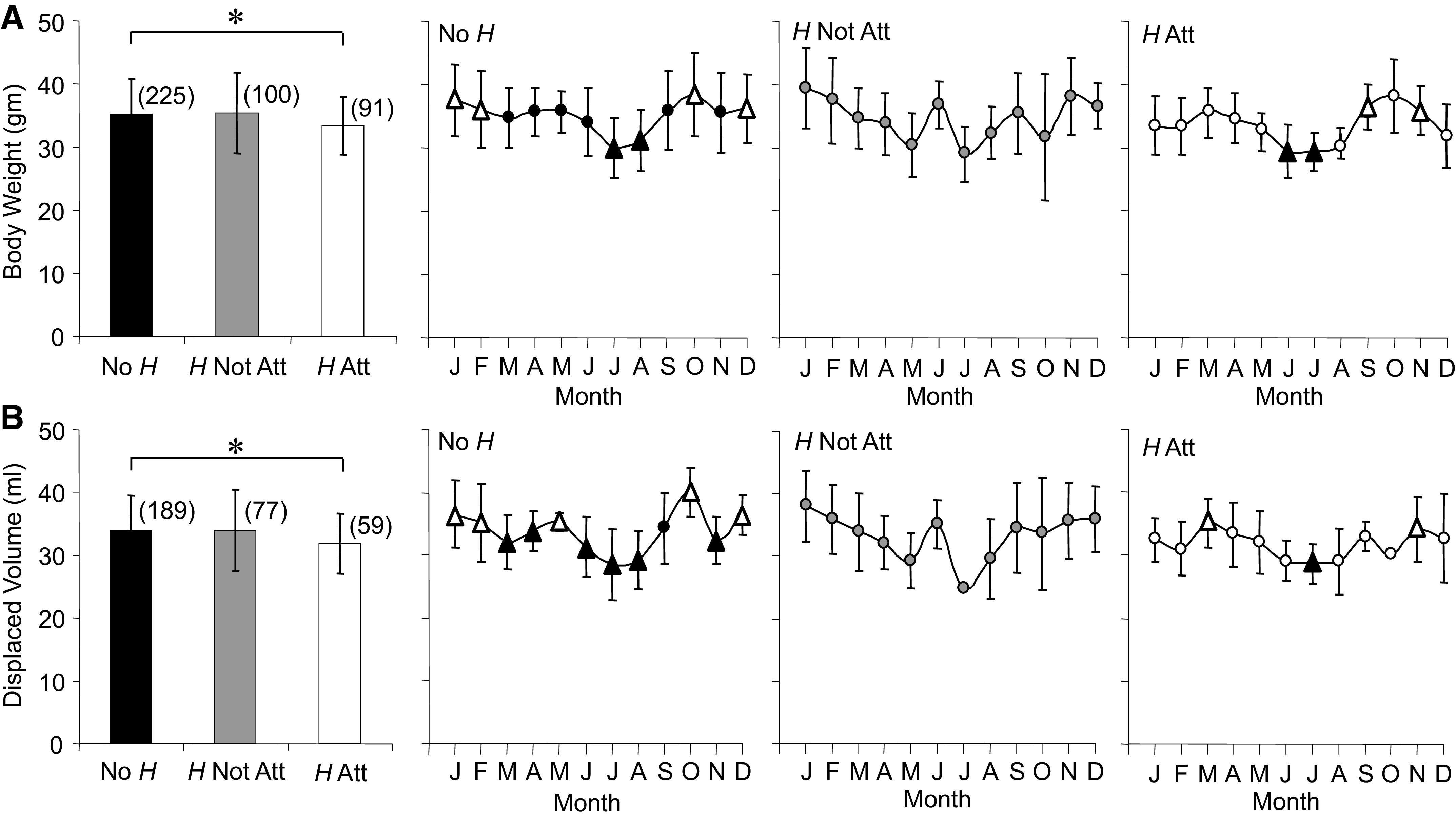
Averages and annual time series for body weight (*A*) and displaced volume (*B*) measured in *Rana pipiens* with three parasite conditions: no *Hematoloechus* in the lungs (No *H*), *Hematoloechus* not attached to the inner lung wall (*H* Not Att), *Hematoloechus* attached to the inner lung wall (*H* Att). Data are presented as means ± SD (*n*). *A*: averages: *No *H* > *H* Att, *P* = 0.02. Time series: no *H*, *P* = 0.0008; *H* Not Att, *P* = 0.01; *H* Att, *P* = 0.001. *B*: averages: *no *H* > *H* Att, *P* = 0.03. Time series: no *H*, *P* < 0.0001; *H* Not Att, *P* = 0.16; *H* Att, *P* = 0.02. Open triangles, different from closed triangles. Months in order: J, January; F, February; M, March; A, April; M, May; J, June; J, July; A, August; S, September; O, October; N, November; D, December.

On average, body weight (Fig. 1*A*) was 2 g lower for *H* Att compared with No *H* and both groups displayed seasonal variation. No *H* body weights were highest in four winter months and lowest in July/August. *H* Att displayed low body weights in summer, similar to No *H*, with highs in late fall and early winter. No *H* body weight decreased at slower rate than its increase, 1.2 versus 3.6 g/month, respectively. Similarly, *H* Att’s body weight rate of change was slower (0.9) compared with the increase (3.6 g/month). Average displaced volume (Fig. 1*B*) was sensitive enough to detect similar changes in average body weight and seasonal changes for No *H* and *H* Att.

**Figure 2.**
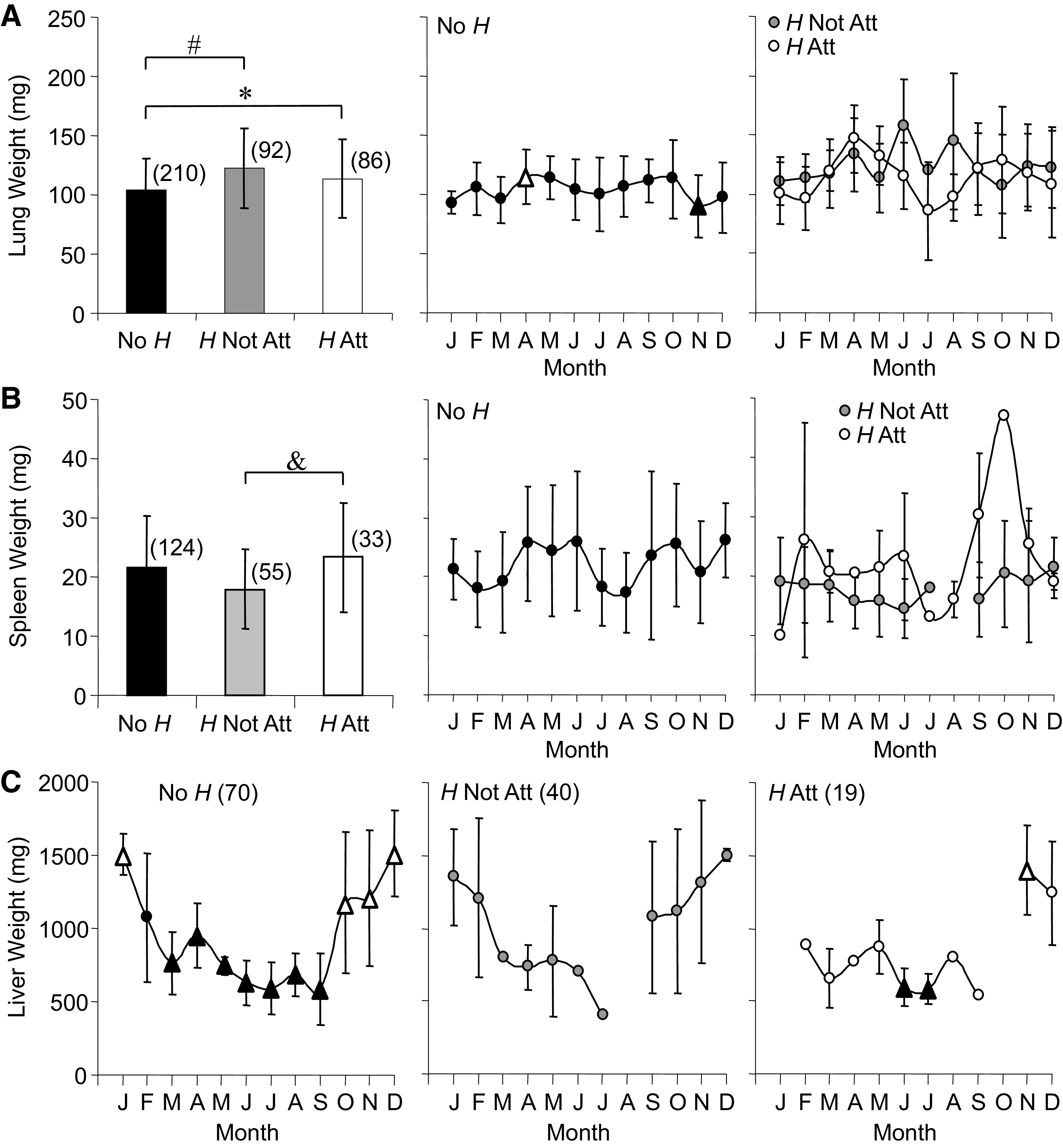
Averages and annual time series for lung (*A*), spleen (*B*), and liver (*C*) weights measured in *Rana pipiens* with three parasite conditions: no *Hematoloechus* in the lungs (No *H*), *Hematoloechus* not attached to the inner lung wall (*H* Not Att), *Hematoloechus* attached to the inner lung wall (*H* Att). Data are presented as means ± SD (*n*). *A*: averages: *No *H* < *H* Att, #No *H* < *H* Not Att (*P* < 0.0001). Times series: No *H*, *P* = 0.02; *H* Not Att, *P* = 0.34; *H* Att, *P* = 0.02. *B*: averages: &*H* Not Att < *H* Att (*P* = 0.006). Time series: no *H*, *P* = 0.12; *H* Not Att, *P* = 0.95; *H* Att, *P* = 0.11. *C*: time series: No *H*, *P* < 0.0001, *H* Not Att, *P* = 0.12; *H* Att, *P* = 0.04. Open triangles, significantly different from closed triangles. Months in order: J, January; F, February; M, March; A, April; M, May; J, June; J, July; A, August; S, September; O, October; N, November; D, December.

After removal of *H* during necropsy, lung weight (Fig. 2*A*) averaged 16% higher for *H* Not Att and 8% higher for *H* Att compared with No *H*. No *H* lung weight varied naturally with the season, highest in April and lowest in November, with a rate of change slower for the decrease than the increase, −3.6 and 5.0 g/month, respectively. Significant seasonal changes in lung weight were not detected for *H* Not Att or *H* Att.

Average spleen weight (Fig. 2*B*) was lower for *H* Not Att compared with *H* Att and neither group differed from No *H*. Distinct oscillations in spleen weight were observed across the year for No *H* and were not present for *H* Not Att or *H* Att. In contrast, No *H* liver weight (Fig. 2*C*) showed a dramatic (257%) seasonal decline (−367.5 g/mo) from its highest average in January (1501.5 SD 139.2 mg) to March, then remained low throughout spring and summer. Liver weight reached its lowest monthly average in early fall (584.0 SD 240.3 mg), then increased into winter at a rate of 588.8 g/month. Both *H* Not Att and *H* Att showed the same downward trend in liver weight from winter into summer as for No *H*. Average liver weights did not differ between groups.

#### Leukocytes.

**Figure 3.**
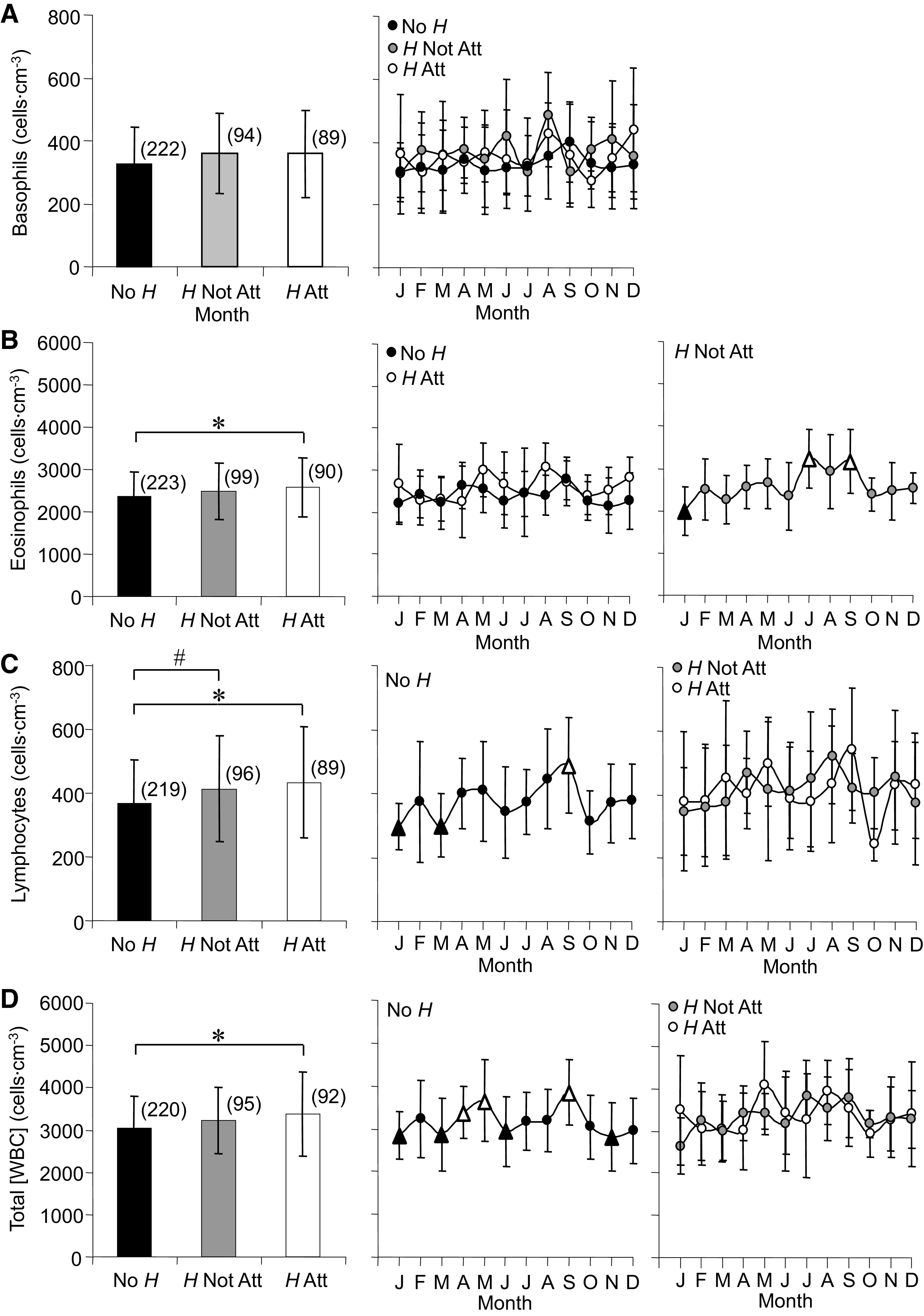
Averages and annual time series for basophils (*A*), eosinophils (*B*), lymphocytes (*C*), and total white blood cell concentration ([WBC], *D*) measured in *Rana pipiens* with three parasite conditions: no *Hematoloechus* in the lungs (No *H*), *Hematoloechus* not attached to the inner lung wall (*H* Not Att), *Hematoloechus* attached to the inner lung wall (*H* Att). Data are presented as means ± SD (*n*). *A*: averages: *P* = 0.02. Time series: No *H*, *P* = 0.77; *H* Not Att, *P* = 0.51; *H* Att, *P* = 0.85. *B*: averages: *No *H* < *H* Att, *P* = 0.01. Time series: No *H*, *P* = 0.03; *H* Not Att, *P* = 0.01; *H* Att, *P* = 0.35. *C*: averages: *No *H* < *H* Att, #No *H* < *H* Not Att, *P* = 0.0009. Time series: No *H*, *P* = 0.001; *H* Not Att, *P* = 0.68; *H* Att, *P* = 0.47. *D*: averages: *No *H* < *H* Att, *P* = 0.003. Time series: No *H*, *P* < 0.0001; *H* Not Att, *P* = 0.22; *H* Att, *P* = 0.53. Open triangles, significantly different from closed triangles. Months in order: J, January; F, February; M, March; A, April; M, May; J, June; J, July; A, August; S, September; O, October; N, November; D, December.

Among the three WBC subtypes, parasite group was a significant factor for average basophil counts (Fig. 3*A*); however, differences between groups could not be determined due to data variability. The time series for basophils did not change across the year.

Average eosinophils (Fig. 3*B*) were higher for *H* Att compared with No *H*. Seasonal variation of eosinophils was detected for *H* Not Att with the lowest monthly average occurring in January and highest in July and September [change rates = −295.7 and 205.5 (cells·cm^−3^)/month, respectively].

Averages for lymphocytes (Fig. 3*C*) were higher for both *H* Not Att and *H* Att compared with No *H*. Seasonal variation occurred in No *H*, with lows in January and March and a high in September [change rates = −40.9 and 25.4 (cells·cm^−3^)/month, respectively].

Similar to eosinophils, average total [WBC] (Fig. 3*D*) were higher for *H* Att compared with No *H*. Similar to lymphocytes, No *H* was the only group that demonstrated seasonal variation in total [WBC], which peaked twice during the year, once in April/May and once in September. Three low months occurred through the winter in January, March, and November, and a fourth low occurred in June (change rates = −459.3 and 302.7 (cells·cm^−3^)/month).

#### Plasma [NOx].

**Figure 4.**
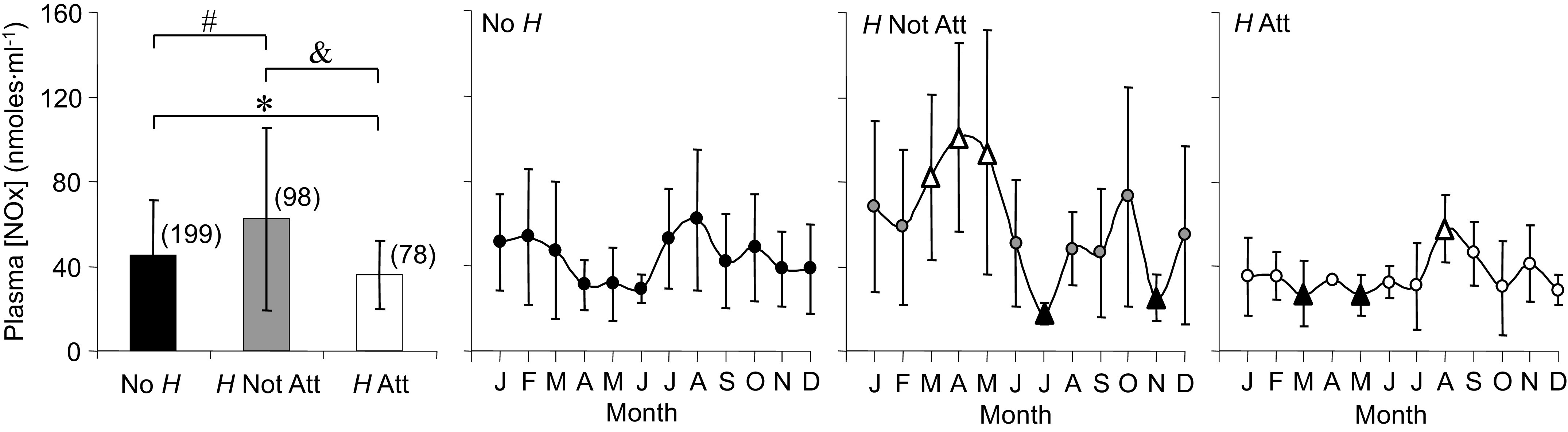
Averages and annual time series for plasma nitrite/nitrate concentration ([NO_x_]) measured in *Rana pipiens* with three parasite conditions: no *Hematoloechus* in the lungs (No *H*), *Hematoloechus* not attached to the inner lung wall (*H* Not Att), *Hematoloechus* attached to the inner lung wall (*H* Att). Data are presented as means ± SD (*n*). Averages: *No *H* > *H* Att; #No *H* < *H* Not Att; &*H* Not Att > *H* Att (*P* < 0.0001). Time series: No *H*, *P* = 0.006; *H* Not Att, *P* = 0.0002; *H* Att, *P* = 0.02. Open triangles, significantly different from closed triangles. Months in order: J, January; F, February; M, March; A, April; M, May; J, June; J, July; A, August; S, September; O, October; N, November; D, December.

Averages for plasma [NO_x_] (Fig. 4) differed among all three groups, highest for *H* Not Att and lowest for *H* Att. Distinct oscillations across the year were apparent for No *H*, and significant seasonal changes between months were identified for the two parasite conditions. For *H* Not Att, plasma [NO_x_] increased dramatically [rate = 14.3 (nmoles·mL^−1^)/month] with a sustained peak in March, April, and May that was threefold higher compared with No *H* during the same timeframe. For *H* Att, [NO_x_] did not peak until August [rate = 10.5 (nmoles·mL^−1^)/month], a peak that was 100% lower (58.1 nmoles·mL^−1^) than the highest monthly average for *H* Not Att (101.1 nmoles·mL^−1^). Troughs occurred in July and November for *H* Not Att [rate = −38.0 (nmoles·mL^−1^)/mo] and March and May for *H* Att [rate = −4.4 (nmoles·mL^−1^)/month], revealing opposite cycles for the two parasite groups. Variability within each month also differed between the three groups. *H* Not Att showed high monthly variability compared with No *H* and was much reduced for *H* Att.

#### Heart rate.

**Figure 5.**
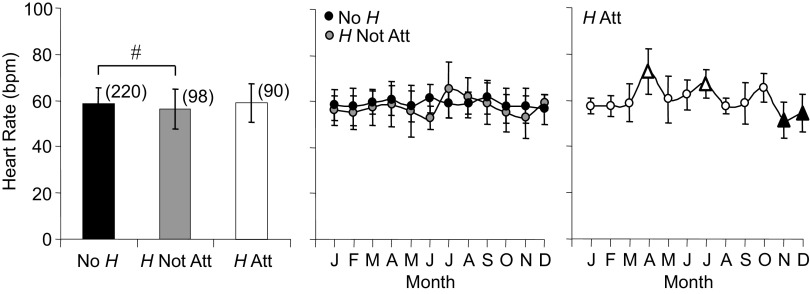
Averages and annual time series for heart rate measured in *Rana pipiens* with three parasite conditions: no *Hematoloechus* in the lungs (No *H*), *Hematoloechus* not attached to the inner lung wall (*H* Not Att), *Hematoloechus* attached to the inner lung wall (*H* Att). Data are presented as means± SD (*n*). Averages: #No *H* > *H* Not Att, *P* = 0.02. Time series: No *H*, *P* = 0.55; *H* Not Att, *P* = 0.34; *H* Att, *P* = 0.0004. Open triangles, different from closed triangles. Months in order: J, January; F, February; M, March; A, April; M, May; J, June; J, July; A, August; S, September; O, October; N, November; D, December.

Figure 5 shows average heart rates that were lower for *H* Not Att versus No *H* and both groups demonstrated steady heart rates throughout the year. In contrast, the average heart rate for *H* Att did not differ from the other two groups; however, seasonal changes in *H* Att were identified, with peaks in April and July and trough in November/December (rate of change between significant monthly averages = 4.5 beats/min/month increase and −3.8 beats/min/month decrease).

#### Blood clotting.

**Figure 6.**
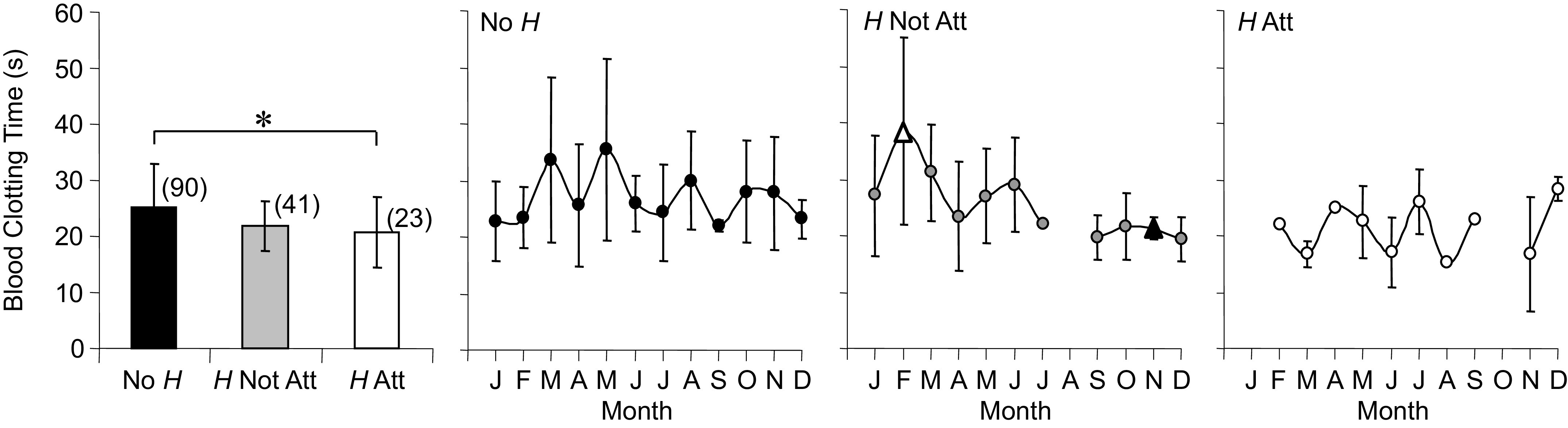
Averages and annual time series for blood clotting time measured in *Rana pipiens* with three parasite conditions: no *Hematoloechus* in the lungs (No *H*), *Hematoloechus* not attached to the inner lung wall (*H* Not Att), *Hematoloechus* attached to the inner lung wall (*H* Att). Data are presented as means ± SD (*n*). Averages: *No *H* > *H* Att (*P* = 0.008). Time series: No *H*, *P* = 0.10; *H* Not Att, *P* = 0.05; *H* Att, *P* = 0.66. Open triangles, significantly different from closed triangles. Months in order: J, January; F, February; M, March; A, April; M, May; J, June; J, July; A, August; S, September; O, October; N, November; D, December.

Figure 6 presents data for time to blood clot formation. No *H* clotting time averaged 5 s longer than *H* Att and demonstrated distinct oscillations across the year with higher month-to-month variability in spring months that diminished as the year progressed. Average clotting time for *H* Not Att did not differ from the other two groups; however, a significant seasonal decline of 17 s from February to December was notable (rates of change = −1.9 and 5.7 s/mo).

#### Erythrocytes.

**Figure 7.**
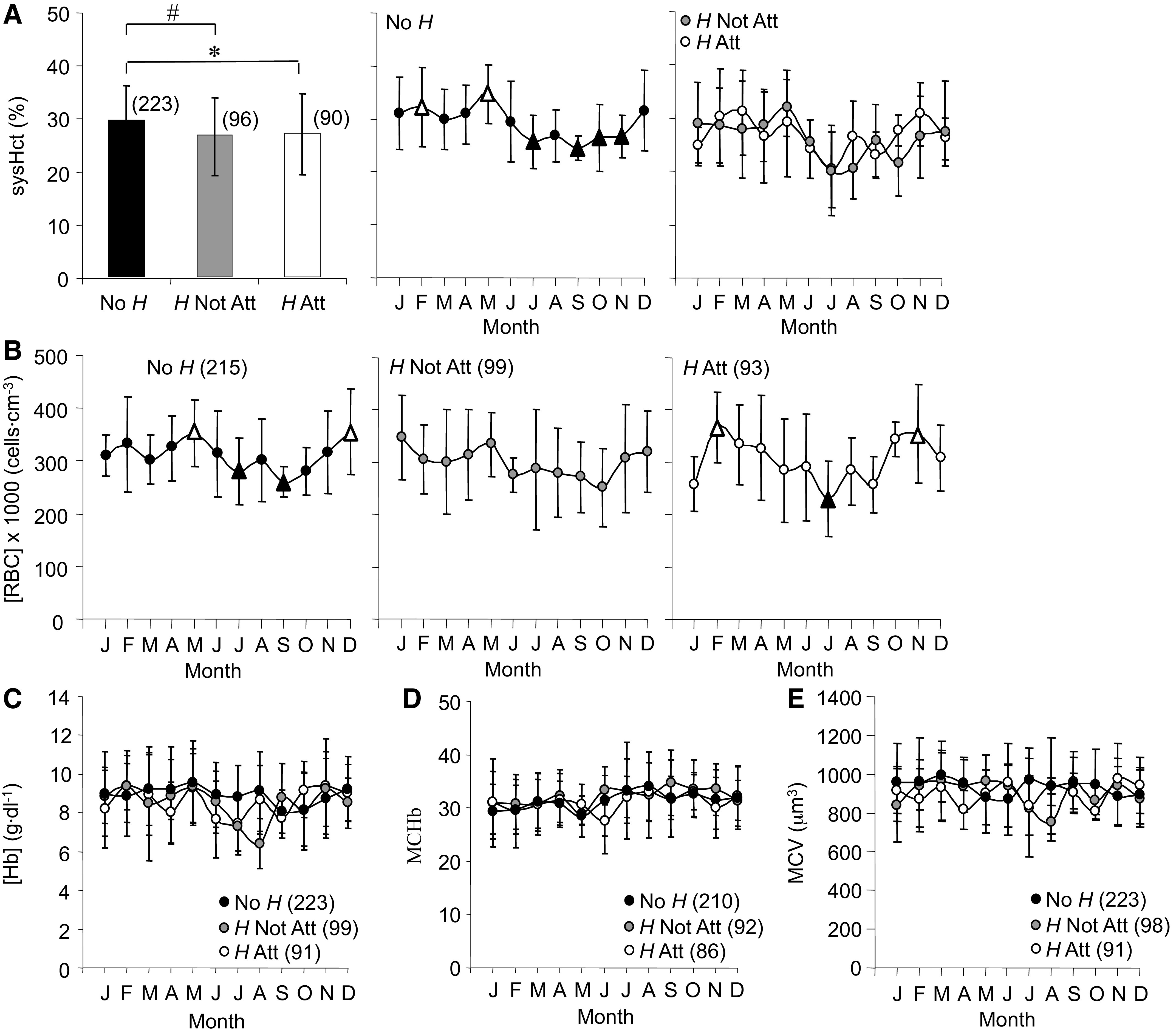
Averages and annual time series for systemic hematocrit (sysHct, *A*), red blood cell concentration ([RBC], *B*), hemoglobin concentration ([Hb], *C*), mean corpuscular hemoglobin (MCHb, *D*), and mean corpuscular volume (MCV, *E*) measured in *Rana pipiens* with three parasite conditions: no *Hematoloechus* in the lungs (No *H*), *Hematoloechus* not attached to the inner lung wall (*H* Not Att), *Hematoloechus* attached to the inner lung wall (*H* Att). Data are presented as means ± SD (*n*). *A*: averages: *No H > H Att, #No *H* > *H* Not Att (*P* = 0.002). Time series: No *H*, *P* < 0.0001; *H* Not Att, *P* = 0.10; *H* Att, *P* = 0.04. *B*: time series: No *H*, *P* = 0.003; *H* Not Att, *P* = 0.60; *H* Att, *P* = 0.006. *C*: time series: No *H*, *P* = 0.74; *H* Not Att, *P* = 0.35; *H* Att, *P* = 0.72. *D*: time series: No *H*, *P* = 0.65; *H* Not Att, *P* = 0.32; *H* Att, *P* = 0.20. *E*: time series: No *H*, *P* = 0.19; *H* Not Att, *P* = 0.75; *H* Att, *P* = 0.92. Open triangles, significantly different from closed triangles. Months in order: J, January; F, February; M, March; A, April; M, May; J, June; J, July; A, August; S, September; O, October; N, November; D, December.

Average sysHct for No *H* was higher than *H* Not Att and *H* Att. No *H* sysHct demonstrated seasonal changes that were highest in late winter and spring and lowest in summer into fall (Fig. 7*A*, rate of change = −4.5 and 1.8%/mo).

Average [RBC] (Fig. 7*B*) did not differ with parasite condition; however, seasonal changes were observed for No *H* [RBC] with peaks in May and December and troughs in July and September [rate of change = −36.4 and 31.9 × 1,000 (cells·cm^−3^)/month], a pattern that was similar to sysHct. *H* Att also showed seasonal changes in [RBC] [rates = −27.2 and 30.8 × 1,000 (cells·cm^−3^)/month] with both peaks shifted earlier in the year compared with No *H*. *H* Att [RBC] declined steadily from a high of 367.3 × 1,000 cells·cm^−3^ in February to 231.1 × 1,000 cells·cm^−3^ in July, a low that was ∼50 × 1,000 cells·cm^−3^ below that of No *H* in the same month (280.8 × 1,000 cells·cm^−3^).

Neither parasite condition nor season influenced [Hb] (Fig. 7*C*), MCHb (Fig. 7*D*), or MCV (Fig. 7*E*).

#### Protein concentrations in three compartments.

**Figure 8.**
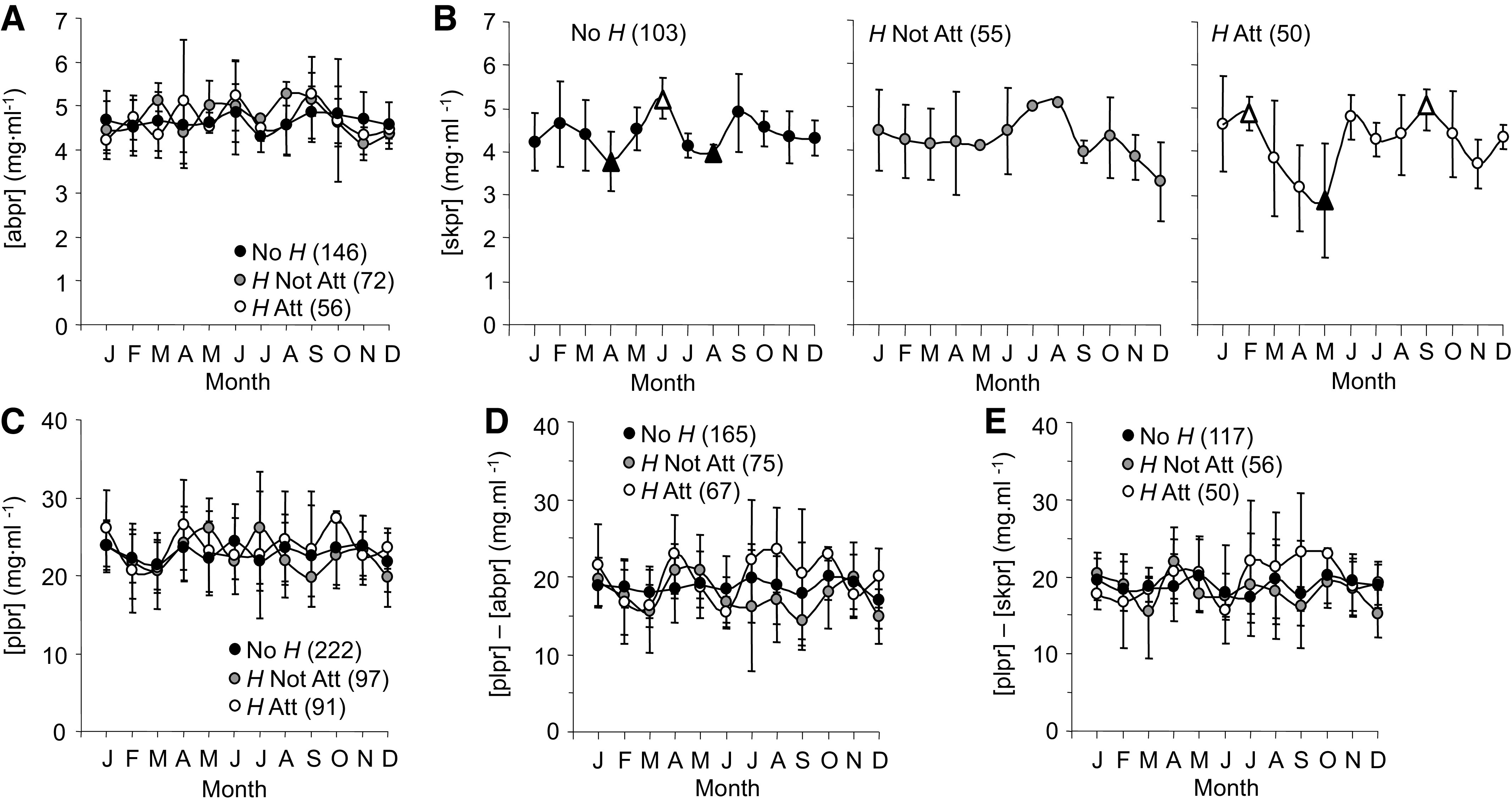
Annual time series for abdominal cavity fluid protein concentration ([abpr], *A*), skin lymph protein concentration ([skpr], *B*), plasma protein concentration ([plpr], *C*), differences between [plpr] and [abpr] (*D*), and differences between [plpr] and [skpr] (*E*) measured in *Rana pipiens* with three parasite conditions: no *Hematoloechus* in the lungs (No *H*), *Hematoloechus* not attached to the inner lung wall (*H* Not Att), *Hematoloechus* attached to the inner lung wall (*H* Att). Data are presented as means ± SD (*n*). *A*: time series: No *H*, *P* = 0.22; *H* Not Att, *P* = 0.16; *H* Att, *P* = 0.42. *B*: time series: No *H*, *P* = 0.82; *H* Not Att, *P* = 0.03; *H* Att, *P* = 0.06. *C*: time series: No *H*, *P* = 0.74; *H* Not Att, *P* = 0.26; *H* Att, *P* = 0.13. *D*: time series: No *H*, *P* = 0.004; *H* Not Att, *P* = 0.64; *H* Att, *P* = 0.009. *E*: time series: No *H*, *P* = 0.91; *H* Not Att, *P* = 0.22; *H* Att, *P* = 0.60. Open triangles, significantly different from closed triangles. Months in order: J, January; F, February; M, March; A, April; M, May; J, June; J, July; A, August; S, September; O, October; N, November; D, December.

Average protein concentrations, [abpr] (abdominal cavity compartment, Fig. 8*A*), [skpr] (compartment between body and skin, Fig. 8*B*), and [plpr] (cardiovascular compartment, Fig. 8*C*) did not differ between groups. Both [abpr] and [plpr] were stable throughout the year. In contrast, [skpr] did vary with season (Fig. 8*B*). Monthly averages for No *H* [skpr] rose by 1.5 mg·mL^−1^ from April to June and fell in August by a similar amount (change rates = −0.7 and 0.8 (mg·mL^−1^)/month, respectively). For *H* Att, [skpr] had two peaks (February and October) that were similar in magnitude compared with No *H*. Importantly, *H* Att [skpr] dropped by almost 50% from 4.9 in February to 2.9 mg·mL^−1^ in May, a low that was well below the lowest No *H* trough average of 3.8 mg·mL^−1^ (rates = −0.7 and 0.5 (mg·mL^−1^)/month, respectively).

Average differences between plasma and abdominal cavity fluid protein ([plpr]–[abpr], Fig. 8*D*) and plasma and skin protein concentrations ([plpr]– [skpr], Fig. 8*E*) were stable across the year, suggesting that colloid osmotic pressure differences at the macro-level were stable between the cardiovascular/abdominal cavity and cardiovascular/skin compartments.

#### Abdominal cavity fluid.

**Figure 9.**
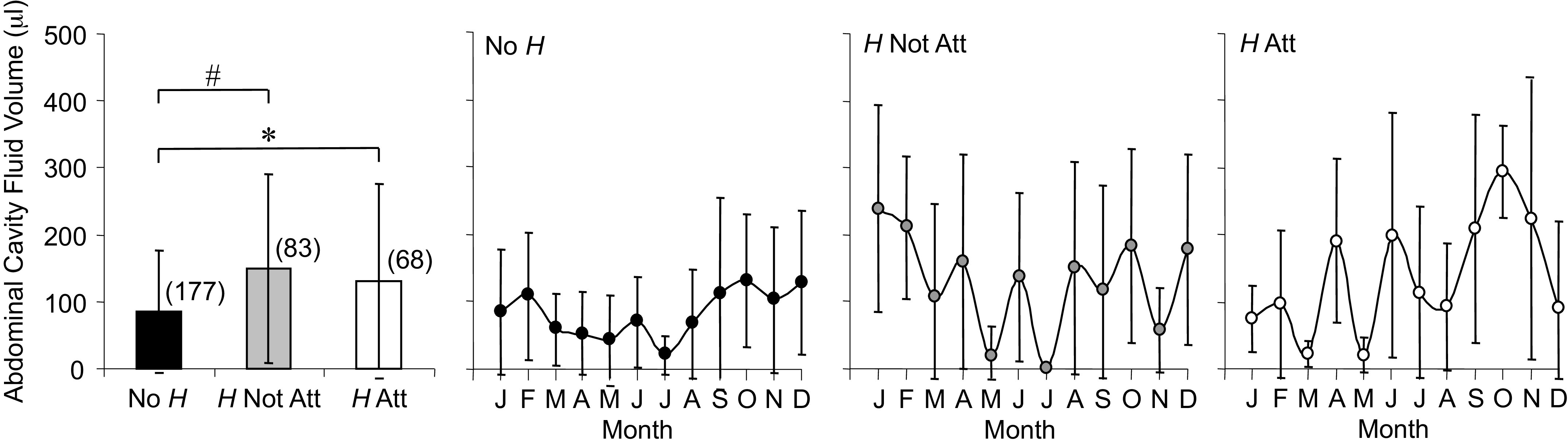
Averages and annual time series for abdominal cavity fluid volume measured in *Rana pipiens* with three parasite conditions: no *Hematoloechus* in the lungs (No *H*), *Hematoloechus* not attached to the inner lung wall (*H* Not Att), *Hematoloechus* attached to the inner lung wall (*H* Att). Data are presented as means ± SD (*n*). Averages: *No *H* < *H* Att, #No *H* < *H* Not Att (*P* = 0.0001). Times series: No *H*, *P* = 0.02; *H* Not Att, *P* = 0.06; *H* Att, *P* = 0.05. Open triangles, significantly different from closed triangles. Months in order: J, January; F, February; M, March; A, April; M, May; J, June; J, July; A, August; S, September; O, October; N, November; D, December.

Average volume of fluid collected from the abdominal cavity (Fig. 9) was greater for *H* Not Att and *H* Att compared with No *H* and varied widely across the year for the two parasite conditions. The stable time series data for [plpr]–[abpr] (Fig. 8*D*) were consistent with macro-level protein concentration differences having minimal impact on the changes in volume of abdominal cavity fluid.

### Capillary Variables

#### Water permeability, hydraulic conductivity.

**Figure 10.**
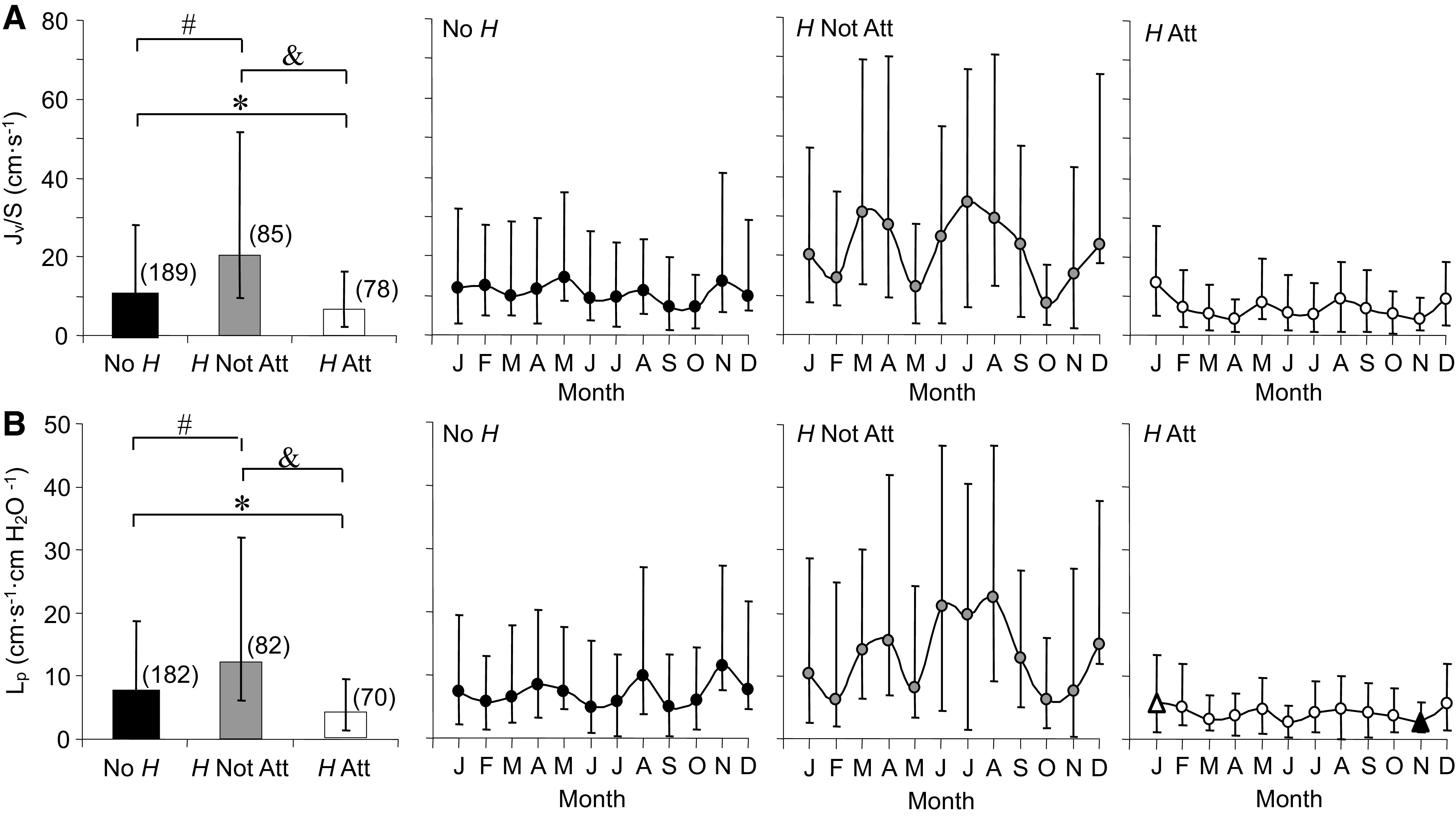
Medians and annual time series for capillary volume flux per surface area (*J*_v_/*S*, *A*) and capillary hydraulic conductivity (*L*_p_, *B*) measured in *Rana pipiens* with three parasite conditions: no *Hematoloechus* in the lungs (No *H*), *Hematoloechus* not attached to the inner lung wall (*H* Not Att), *Hematoloechus* attached to the inner lung wall (*H* Att). Data are median ± 25/75% (*n*). *A*: medians: *No *H* > *H* Att; #No *H* < *H* Not Att; &*H* Not Att > *H* Att (*P* < 0.0001). Time series: No *H*, *P* = 0.41; *H* Not Att, *P* = 0.12; *H* Att, *P* = 0.07. *B*: medians: *No *H* > *H* Att; #No *H* < *H* Not Att; &*H* Not Att > *H* Att (*P* < 0.0001). Time series: No *H*, *P* = 0.52; *H* Not Att, *P* = 0.51; *H* Att, *P* = 0.006. Open triangles, significantly different from closed triangles. Months in order: J, January; F, February; M, March; A, April; M, May; J, June; J, July; A, August; S, September; O, October; N, November; D, December.

For individual capillaries, median *J*_v_/*S* (Fig. 10*A*) and median *L*_p_ (Fig. 10*B*) differed among all three groups, highest for *H* Not Att and lowest for *H* Att. For No *H*, *J*_v_/*S* and *L*_p_ were lower than *H* Not Att and higher than *H* Att. Across the year, *J*_v_/*S* and *L*_p_ showed no significant seasonal changes for No *H* or *H* Not Att. *H* Not Att, in particular, was much more variable from month to month than the other two groups. The error bars show a shift from skewed to normal distributions in *J*_v_/*S* and *L*_p_ data during June, July, and August for *H* Not Att, a shift that was not present for No *H*.

*H* Att was the only group with significant seasonal variation in *L*_p_, which was highest in January, lowest in November [rate = -0.3 and 1.6 (cm·s^−1^·cmH_2_O^−1^)/month], and displayed normal distributions in April, May, August, and September. The Δτ mechanical stimulus for each capillary averaged 29.0 (SD 10.5) dynes·cm^−2^ and did not differ for parasite conditions or season.

**Figure 11.**
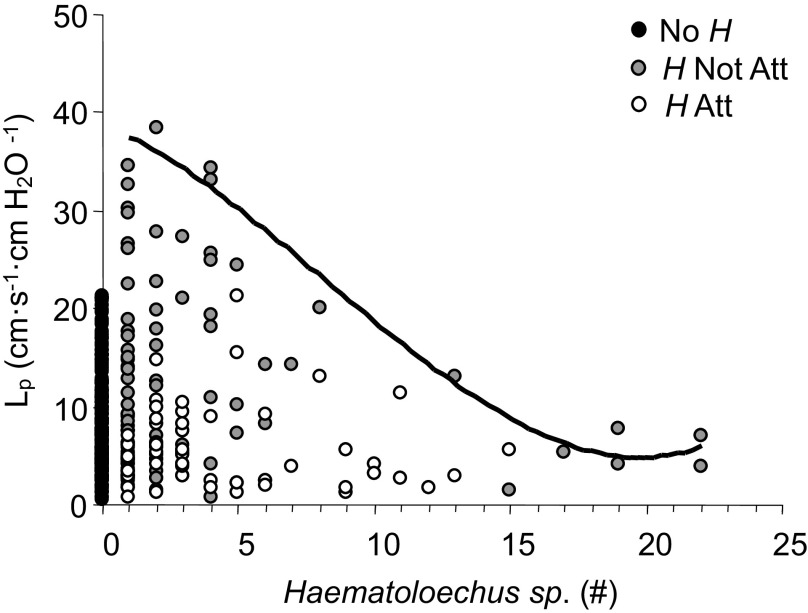
Individual values of capillary hydraulic conductivity (*L*_p_) plotted as a function of number of *Hematoloechus* measured in *Rana pipiens* with three parasite conditions: no *Hematoloechus* in the lungs (No *H*), *Hematoloechus* not attached to the inner lung wall (*H* Not Att), *Hematoloechus* attached to the inner lung wall (*H* Att). Bold, solid line is the dose/response curve formed by the data limit (*R*^2^ = 0.98).

Figure 11 illustrates the relationship between the number of *H* and *L*_p_. The data displayed a dose/response curve at the limit with a negative slope that extended from a high value of 38.3 to a low value of 3.9 × 10^−7^ cm·s^−1^·cmH_2_O^−1^. *H* Not Att accounted for the highest values of *L*_p_ shown under the curve and approached the lower, *H* Att values of *L*_p_, as the number of *H* increased.

#### Balance, σ(π_c_–π_i_), and burst pressures.

**Figure 12.**
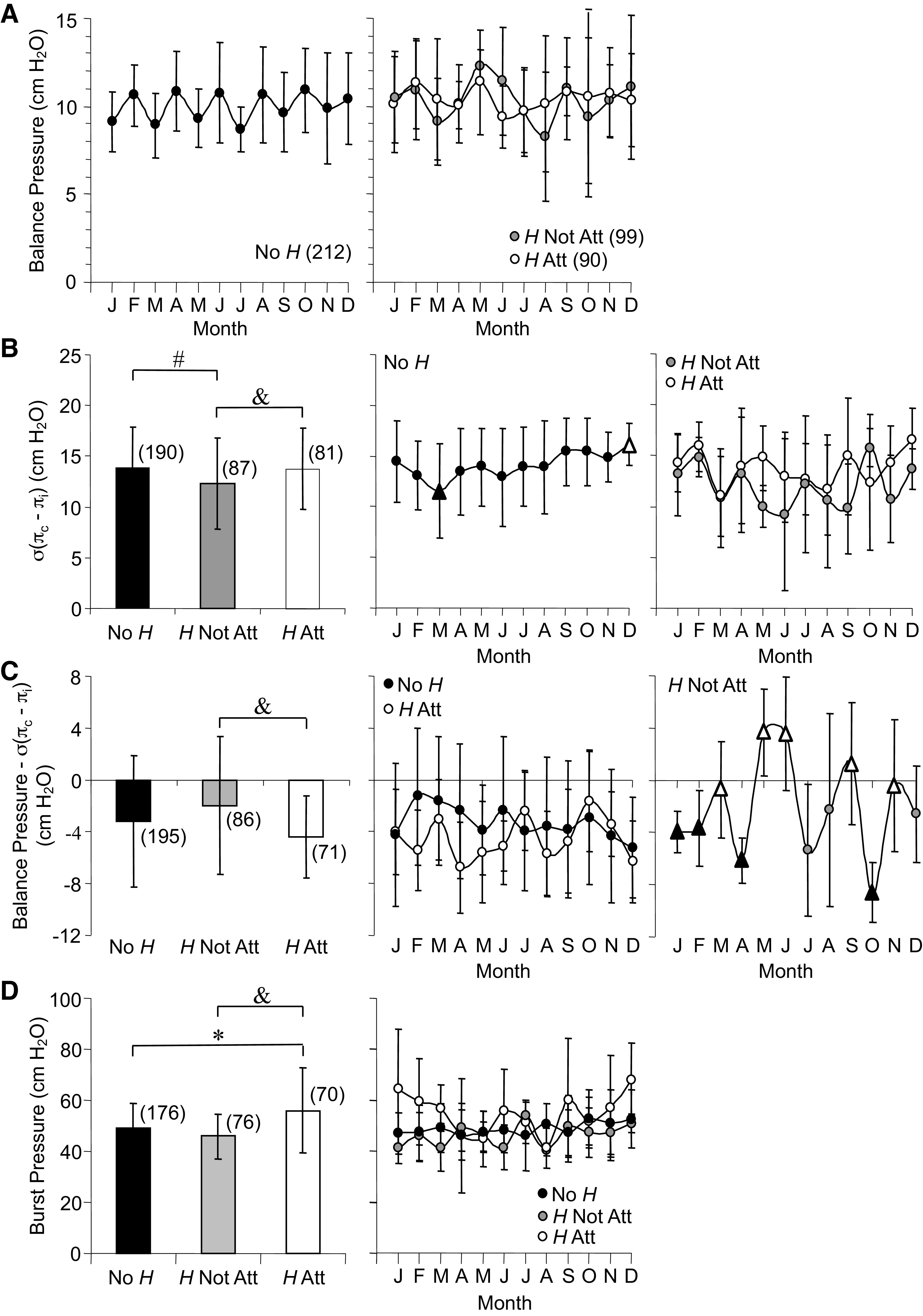
Averages and annual time series for capillary balance pressure (*A*), capillary sigma delta pi [σ(π_c_–π_i_), *B*], capillary balance pressure minus sigma delta pi [σ(π_c_–π_i_), *C*], and capillary burst pressure (wall tensile strength, *D*) measured in *Rana pipiens* with three parasite conditions: no *Hematoloechus* in the lungs (No *H*), *Hematoloechus* not attached to the inner lung wall (*H* Not Att), *Hematoloechus* attached to the inner lung wall (*H* Att). Data are presented as means ± SD (*n*). Note differences between *y*-axes. *A*: time series: No *H*, *P* = 0.01; *H* Not Att, *P* = 0.53; *H* Att, *P* = 0.94. *B*: averages: #No *H* > *H* Not Att; &*H* Att > *H* Not Att, *P* = 0.007. Time series: No *H*, *P* = 0.03; *H* Not Att, *P* = 0.13; *H* Att, *P* = 0.28. *C*: averages: &*H* Not Att < *H* Att, *P* < 0.007. Time series: No *H*, *P* = 0.37; *H* Not Att, *P* < 0.0001; *H* Att, *P* = 0.31. *D*: averages: *H Att > No *H*; &*H* Att > *H* Not Att, *P* < 0.0001. Time series: No *H*, *P* = 0.65; *H* Not Att, *P* = 0.39; *H* Att, *P* = 0.34. Open triangles, significantly different from closed triangles. Months in order: J, January; F, February; M, March; A, April; M, May; J, June; J, July; A, August; S, September; O, October; N, November; D, December.

The three pressure variables for individual capillaries are presented in Fig. 12. First, balance pressure (Fig. 12*A*) is the pressure at which flow through the capillary tube from proximal to distal end is zero. Balance pressure did not differ between groups. Time across the year, however, did influence No *H* balance pressure, which oscillated around an average of 10.0 cmH_2_O. In contrast, absence of regular oscillations was the most notable feature of the balance pressure time series data for *H* Not Att and *H* Att.

Figure 12*B* shows the second capillary pressure, σ(π_c_–π_i_), which is the *x*-axis intercept of the pressure/*J*_v_/*S* plots used to assess *L*_p_ and the pressure at which filtration flow through the capillary wall is zero. Average σ(π_c_–π_i_) was lowest for *H* Not Att compared with both No *H* and *H* Att. A distinct seasonal pattern was revealed for No *H* where σ(π_c_–π_i_) was lowest in March, increased steadily to a peak in December at a rate of 0.5 cmH_2_0/month, and declined at a faster rate (−1.5 cmH_2_O/month) back to March. For the two parasite groups, the annual time course of σ(π_c_–π_i_) was more variable and no seasonal changes were detected.

Average differences between balance pressure and σ(π_c_–π_i_) pressure are shown in Fig. 12*C.* Overall, *H* Not Att was less than *H* Att. *H* Not Att displayed seasonal variation with average difference between the two pressures rising above zero in May, June, and September, an increase that would favor flow through the tissue during those months if precapillary resistance remained constant. In contrast, average pressure difference for No *H* and *H* Att remained negative throughout the year, which would favor filtration into tissues if resistance upstream was constant.

Among the 38 variables in this study, the third, capillary burst pressure (wall tensile strength, Fig. 12*D*), was the only study variable where the average for *H* Att was higher than both No *H* and *H* Not Att. Time series data showed consistent wall strength across the year for No *H* and *H* Not Att. In contrast, capillaries in *H* Att displayed burst pressures that varied month-to-month and by as much as 20 cmH_2_O between summer and winter.

#### Rheology.

**Figure 13.**
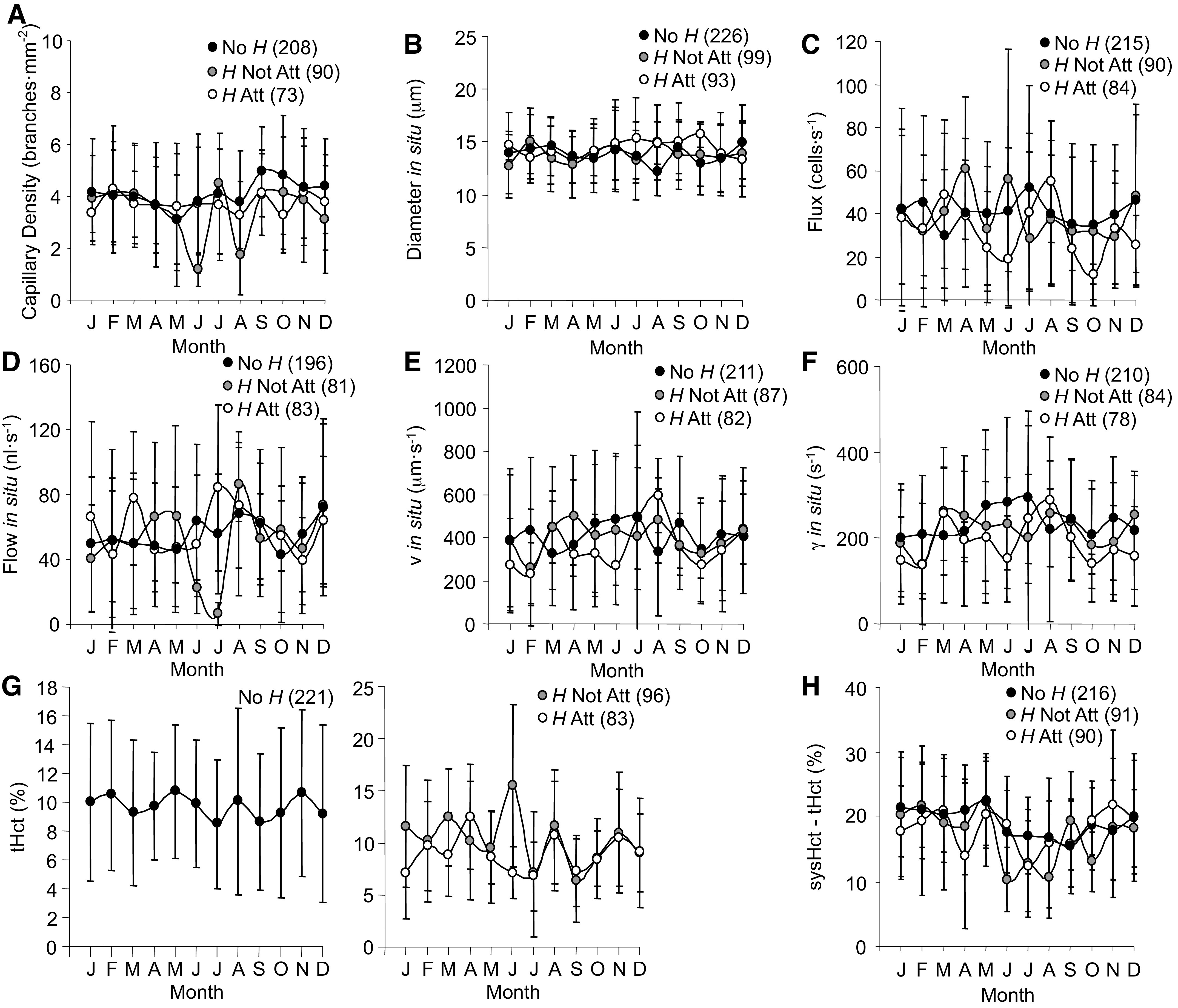
Annual time series for capillary density (*A*), capillary diameter (*B*), red blood cell (RBC) flux (*C*), RBC flow (*D*), RBC mean velocity (*v*, *E*), RBC shear rate (γ, *F*), tube hematocrit (tHct, *G*), and delta hematocrit (sysHct–tHct, *H*) measured in *Rana pipiens* with three parasite conditions: no *Hematoloechus* in the lungs (No *H*), *Hematoloechus* not attached to the inner lung wall (*H* Not Att), *Hematoloechus* attached to the inner lung wall (*H* Att). Data are presented as means ± SD (*n*). *A*: time series: No *H*, *P* = 0.60; *H* Not Att, *P* = 0.21; *H* Att, *P* = 1.00. *B*: time series: No *H*, *P* = 0.56; *H* Not Att, *P* = 0.90; *H* Att, *P* = 0.95. *C*: time series: No *H*, *P* = 0.89; *H* Not Att, *P* = 0.78; *H* Att, *P* = 0.37. *D*: time series: No *H*, *P* = 0.75; *H* Not Att, *P* = 0.64; *H* Att, *P* = 0.41. *E*: time series: No *H*, *P* = 0.77; *H* Not Att, *P* = 0.95; *H* Att, *P* = 0.05. *F*: time series: No *H*, *P* = 0.68; *H* Not Att, *P* = 0.79; *H* Att, *P* = 0.16. *G*: time series: No *H*, *P* = 0.97; *H* Not Att, *P* = 0.36; *H* Att, *P* = 0.48. *H*: time series: No *H*, *P* = 0.13; *H* Not Att, *P* = 0.23; *H* Att, *P* = 0.69. Months in order: J, January; F, February; M, March; A, April; M, May; J, June; J, July; A, August; S, September; O, October; N, November; D, December.

The comprehensive set of capillary measures in this study included two structure variables. First, density of the mesenteric capillary network did not differ among parasite conditions or seasons (Fig. 13*A*). Second, capillary diameter also did not differ among parasite conditions and remained steady throughout the year (Fig. 13*B*). Likewise, none of the indices of capillary oxygen delivery were impacted by parasite or season. RBC flux (Fig. 13*C*) and RBC flow (Fig. 13*D*) were steady. RBC *v* (Fig. 13*E*) tended to rise in summer, although not significantly, and γ (Fig. 13*F*) reflected *v* rather than diameter. tHct (Fig. 13*G*) oscillated, but did not differ statistically between groups or across the year. sysHct minus tHct (Fig. 13*H*), while not different between groups, did trend lower in summer, reflecting the seasonal changes in sysHct (Fig. 7*A*).

### Capillary *L*_p_ Outliers—Secondary Analysis

#### No hematoloechus.

Median (± 25/75%) control *L*_p_ was 7.0 (± 4.3/11.3, *n* = 182) versus 33.0 (± 27.3/59.3, *n* = 28) × 10^−7^ cm·s^−1^·cmH_2_O^−1^ for *L*_p_ outliers in the No *H* group. Of the 38 variables tested, five systemic and four capillary variables distinguished the animals with *L*_p_ outliers from control. Basophils, eosinophils, lymphocytes, total [WBC], clotting time, capillary σ(π_c_–π_i_), and capillary burst pressure were lower, and balance pressure minus σ(π_c_–π_i_) was significantly higher for the *L*_p_ outliers versus control (Table 1).

**Table 1.** Systemic and capillary variables measured in Rana pipiens for three parasite conditions

	Control	*L*_p_ Outliers	*P*
No *H*			
*L*_p_, median, cm·s^−1^·cmH_2_O^−1^	7.0 × 10^−7^	33.0 × 10^−7^	
Basophils, cells·cm^−3^	350 (± 153) (182)	289 (± 93) (28)	0.005
Eosinophils, cells·cm^−3^	2,463 (± 648) (182)	2,107 (± 633) (28)	0.0007
Lymphocytes, cells·cm^−3^	405 (± 189) (182)	327 (± 132) (28)	0.009
Total [WBC], cells·cm^−3^	3,229 (± 880) (182)	2,723 (± 714) (28)	0.002
Clotting time, s	30.4 (± 15.0) (84)	22.8 (± 11.9) (14)	0.05
** σ**(π_c_–π_i_), cmH_2_O	13.7 (± 4.5) (171)	11.7 (± 4.6) (27)	0.03
Burst pressure, cmH_2_O	54.6 (± 16.6) (157)	43.1 (± 8.5) (23)	<0.0001
Balance pressure, σ(π_c_–π_i_) − cmH_2_O	−3.2 (± 5.4) (171)	−0.9 (± 6.0) (27)	0.04
*H* Not Att			
* L*_p_, median, cm·s^−1^·cmH_2_O^−1^	12.4 × 10^−7^	68.0 × 10^−7^	
Capillary density, branches·mm^−2^	3.9 ± 2.6 (75)	2.2 ± 1.8 (8)	0.03
[plpr], mg·mL^−1^	22.1 ± 4.3 (80)	25.6 ± 6.7 (8)	0.04
*H* Att			
* L*_p_, median, cm·s^−1^·cmH_2_O^−1^	4.2 × 10^−7^	20.0 × 10^−7^	
Spleen weight, mg	28.2 (± 15.0) (24)	18.1 (± 7.8) (7)	0.03
Balance pressure, cmH_2_O	10.9 (± 3.2) (70)	9.0 (± 1.3) (18)	0.0002

Data are means (SD) (*n*). Animals were grouped as control or outliers based on statistical analyses of capillary *L*_p_ data. No *H*, no *Hematoloechus* in the lungs; *H* Not Att, not attached to the inner lung wall; *H* Att, *Hematoloechus* attached to the inner lung wall; *L*_p_, capillary hydraulic conductivity; [plpr], plasma protein concentration; σ(π_c_–π_i_), capillary sigma delta pi; [WBC], white blood cell concentration.

#### Hematoloechus not attached.

Median (± 25/75%) control *L*_p_ was 12.4 (± 6.2/19.6, *n* = 82) versus 68.0 (± 53.2/79.3, *n* = 8) × 10^−7^ cm·s^−1^·cmH_2_O^−1^ for the *L*_p_ outliers in the *H* Not Att group. In contrast to No *H*, only capillary density and [plpr] distinguished control from *L*_p_ outliers for *H* Not Att (Table 1).

#### Hematoloechus attached.

Median (± 25/75%) control *L*_p_ was 4.2 (± 2.7/5.5, *n* = 70) versus 20.0 (± 13.5/33.4, *n* = 18) × 10^−7^ cm·s^−1^·cmH_2_O^−1^ for *L*_p_ outliers. The variables that were unique to *H* Att were spleen weight and capillary balance pressure. Both were lower for *H* Att *L*_p_ outliers compared with No H (Table 1).

## DISCUSSION

The present prospective, descriptive study had two primary objectives: *1*) to determine the impact of *Hematoloechus* sp. (*H*) on *Rana pipiens* and *2*) to establish baselines across the year for a broad range of systemic and individual capillary variables. The first of the two study objectives revealed that 47% of the 38 descriptive, cardiovascular, and immunological variables showed one or more significant changes in *Rana pipiens* with *H*. In time, we recognized that *H* attachment status was an important effector and, ultimately, three groups were identified: no lung *Hematoloechus* (No *H*), *Hematoloechus* not attached in the lungs (*H* Not Att), and *Hematoloechus* attached to the inner lung surface (*H* Att). The second objective indicated previously unknown, naturally occurring seasonal changes for 10 variables in No *H*, 3 in *H* Not Att, and 8 in *H* Att. Chronic diseases of inflammation, parasitic diseases, and stroke are among the significant clinical applications of the study. Past, present, and future studies of capillary physiology using *Rana pipiens* are among the basic sciences expected to benefit from the data.

### Overall Impact of Hematoloechus (Parasite) on Rana Pipiens (Host)

Table 2 provides a succinct snapshot summary of the broad in vivo effects that *H* had on *Rana pipiens*. Ten combinations of differences between average values were discovered among the variables across the three parasite conditions. Of note among those listed is a cluster of eight pro-inflammatory indicators in *H* Not Att—*1*) elevated lung weight, *2*) elevated abdominal cavity fluid volume, and *3*) decreased heart rate, all of which were secondary to *4*) higher capillary *J*_v_/*S*, *5*) higher capillary *L*_p_, and *6*) lower capillary σ(π_c_–π_i_), and, in turn, secondary to *7*) elevated lymphocytes and *8*) elevated plasma [NO_x_]. Of interest, lymphocytes were the only leukocyte subtype that increased and remained elevated in both parasite groups. Basophils had a statistically significant parasite effect; however, differences between groups could not be distinguished, and eosinophils were elevated only in the *H* Att group. Thus, it appears that the host response in *H* Not Att was specific enough to blunt or inhibit parasite-induced leukocyte responders (basophils and eosinophils), but unable to prevent lymphocytes and/or plasma [NO_x_] from triggering the array of inflammation indicators listed above.

**Table 2.** Summary of variables with significant differences between one or more groups of lung parasite conditions in Rana pipiens

Group Differences	Variable	Figure
No *H* > *H* Not Att	Heart rate	Fig. 5
No *H* < *H* Not Att	Plasma [NO_x_], *J*_v_/*S*, *L*_p_	Figs. 4, 10*A*, 10*B*
No *H* > *H* Att	Body weight, displaced volume, plasma [NO_x_], clotting time, *J*_v_/*S*, *L*_p_	Figs. 1*A*, 1*B*, 4, 6, 10*A*, 10*B*
No *H* < *H* Att	Eosinophils, total [WBC]	Figs. 3*B*, 3*D*
* H* Not Att > *H* Att	Plasma [NO_x_], *J*_v_/*S*, *L*_p_, balance pressure − σ(π_c–_π_i_)	Figs. 4, 10*A*, 10*B*, 12*C*
* H* Not Att < *H* Att	Spleen weight	Fig. 2*B*
No *H* > *H* Not Att and *H* Att	sysHct	Fig. 7*A*
No *H* < *H* Not Att and *H* Att	Lung weight, lymphocytes, abdominal cavity fluid volume	Figs. 2*A*, 3*C*, 9
No *H* and *H* Att > *H* Not Att	σ(π_c_–π_i_)	Fig. 12*B*
No *H* and *H* Not Att < *H* Att	Burst pressure	Fig. 12*D*

Data are presented in the figures listed. No *H*, no *Hematoloechus in the lung*; *H* Not Att, *Hematoloechus* not attached; *H* Att, *Hematoloechus* attached to the inner surface of the lungs; *J*_v_/*S*, volume flux per surface area; *L*_p_, hydraulic conductivity; [NO_x_], nitrite/nitrate concentration; sysHct, systemic hematocrit; σ(π_c_–π_i_), sigma delta pi; [WBC], white blood cell concentration.

Blood clotting is also associated with inflammation. Here, eosinophils, total [WBC], and coagulation rate differed in the *H* Att. The average coagulation rate was fastest, and eosinophils and total [WBC] were elevated compared with No *H*. These observations plus the fact that pro-inflammatory indicators (lung weight, abdominal cavity fluid volume, and lymphocytes) were elevated in *H* Not Att, but did not resolve with *H* attachment suggest that the presence of unattached *H* induced symptoms of inflammation that were sustained upon *H* attachment and that eosinophils, total [WBC], and coagulation responded subsequently to *H* attachment.

A very important distinction between the two parasite groups was that inflammation in *H* Att reversed the increases in plasma [NO_x_] and capillary *L*_p_ that were observed in *H* Not Att. *H* Att had the lowest average plasma [NO_x_], which not only fell below *H* Not Att, but also was significantly lower than No *H*. Although mechanism/s for the dramatic changes in [NO_x_] are not known, it appears that the ubiquitous nitric oxide molecule was involved with both proinflammatory and anti-inflammatory processes, all associated with the *H* parasite and its attachment status. Sources of plasma [NO_x_] include endothelial, immune, and nervous system cells. An important aim for future investigations would be to discover how *H* increases and decreases nitric oxide in *Rana pipiens* and if these outcomes are achieved dose dependently.

Regarding *L*_p_, *H* Att capillaries were “tighter” (lower *L*_p_) than *H* Not Att and, surprisingly, also tighter than No *H*, which would be considered an anti-inflammatory phenotype profile for capillaries. It appears that *H* attachment resulted in parasite-host biochemical communication that not only bypassed or counteracted inflammatory processes at the capillary level of the cardiovascular system, but actually lowered median *L*_p_ by more than 100% below that for No *H*. The number of *H*/*L*_p_ dose/response curve further supported a correlation between *H* and capillary *L*_p_. Higher capillary burst pressure, an index of wall tensile strength, also occurred with *H* attachment and did not change in parallel with changes in capillary *L*_p_. Capillary wall strength for No *H* and *H* Not Att remained stable in the face of dynamic changes in *L*_p_ reflecting two separate sets of molecular machinery acting in parallel—one that maintains wall strength and is not sensitive to *H* and a second that induces a wide range of changes in *L*_p_ and is sensitive to *H*. Mechanisms for these results are unknown at this time. Investigative attention to this important distinction is warranted.

Overall, organ and body weights provided useful indicators of the additive effects of *H* on the host. Fluid accumulated in the lungs, abdominal cavity, and cardiovascular space (lower hematocrit) of *H* Not Att and the excess fluid (organ weight) did not resolve with *H* attachment. Higher levels of organ fluid for both parasite groups in the face of opposing barrier functions suggested two different mechanisms leading to increased organ fluid/weight based on *H* attachment status: *1*) excess fluid filtered into tissues and compartments via leaky capillaries (*H* Not Att) and *2*) fluid trapped in tissues and compartments via tight capillaries (*H* Att). Of the two mechanisms that accounted for similar fluid accumulation in organs, cavities, and spaces, only the “tight” capillaries in *H* Att would trap fluid and also restrict nutrients. In actual fact, average body weight was lower only for *H* Att, a consequence that likely was due to diminished nutrition secondary to low permeability.

### Seasonal Variation

The catalogue of baseline information on *Rana pipiens* that was created from measurements performed across the calendar year for all 38 variables is discussed below and organized according to the three parasite conditions. In many cases, waveforms were identified in the times series data and these are summarized in Table 3. The discussion below highlights comparisons between variables based on similar waveforms and seasonal changes. The data provide valuable insight into the presence of natural internal clocks for the host and parasite.

**Table 3.** Summary of waveforms and associated variables observed in annual time series data for three groups of lung parasite conditions in Rana pipiens

Waveform	Variable	Figure
No *H*		
One oscillation/yr	Body weight, sysHct, [RBC], [skpr], abdominal cavity fluid volume	Figs. 1*A*, 7*A*, 7*B*, 8*B*, 9
Two oscillations/yr	Spleen weight, total [WBC], plasma [NO_x_]	Figs. 2*B*, 3*D*, 4
Four oscillations/yr	Clotting time, *L*_p_, tHct	Figs. 6, 10*B*, 13*G*
Five oscillations/yr	Balance pressure	Fig. 12*A*
Square wave	Liver weight	Fig. 2*C*
Single saw tooth	Lymphocytes, σ(π_c_–π_i_), Balance pressure − σ(π_c_–π_i_)	Figs. 3*C*, 12*B*, 12*C*
Flat line	Eosinophils, heart rate, [Hb], MCHb, MCV, [abpr], [plpr], delta protein concentrations, Burst pressure, diameter	Figs. 3*B*, 5, 7*C*, 7*D*, 7*E*, 8*A*, 8*C*, 8*D*, 8*E*, 12*D*, 13*B*
*H* Not Att		
Two oscillations/yr	Plasma [NO_x_]	Fig. 4
Three oscillations/yr	*J*_v_/*S*, *L*_p_	Figs. 10*A*, 10*B*
Four oscillations/yr	Balance pressure − σ(π_c_–π_i_)	Fig. 12*C*
Saw tooth	Eosinophils, clotting time	Figs. 3*B*, 6
*H* Att		
One oscillation/yr	Body weight, plasma [NO_x_], heart rate, [skpr]	Figs. 1*A*, 4, 5, 8*B*
Two oscillations/yr	[RBC]	Fig. 7*B*
Three oscillations/yr	Balance pressure − σ(π_c_–π_i_)	Fig. 12*C*
Four oscillations/yr	Abdominal cavity fluid volume	Fig. 9
Saw tooth	*L*_p_	Fig. 10*B*

Data are presented in the figures listed. No *H, Haematoloechus*; *H* Not Att, *Haematoloechus* not attached; *H* Att, *Haematoloechus* attached to the inner surface of the lungs; [Skpr], skin lymph fluid protein concentration; sysHct, systemic hematocrit; [RBC], red blood cell concentration; [WBC], white blood cell concentration; Plasma [NOx], nitrite/nitrate concentration; tHct, capillary tube hematocrit; *L*_P_, capillary hydraulic conductivity; σ(π_c_–π_i_), capillary sigma delta pi; [Plpr], plasma protein concentration; [Abpr], abdominal cavity fluid protein concentration; [Hb], hemoglobin concentration; MCHb, mean corpuscular hemoglobin; MCV, mean corpuscular volume; *J*_v_/*S*, capillary volume flux per surface area.

#### Rana pipiens without lung hematoloechus (No H).

One complete oscillation cycle across the year was observed most frequently in the No *H* dataset. Best examples were the time series traces for sysHct (Fig. 7*A*, No *H*) and [RBC] (Fig. 7*B*, No *H*). Both curves were similar with peaks in winter/spring and troughs in summer, suggesting that changes in RBC, not plasma volume, were causal of sysHct changes. Capillary γ (Fig. 13*F*) trended toward a similar pattern 1 month ahead of drops in sysHct and [RBC], a response that was consistent with lower resistance to flow in the microcirculation occurring ahead of detectable system level changes in sysHct and [RBC].

Double-peaked oscillations were identified for spleen weight (Fig. 2*B*, No *H*), total [WBC] (Fig. 3*D*, No *H*), and plasma [NO_x_] (Fig. 4, No *H*). The spleen is the main erythropoietic organ in *Rana pipiens* (25). Nitric oxide has been shown to impact fluid extravasation from the splenic circulation in rats (26) and heme-maturation of hemoglobin at low doses in mice (27). Here, the frequencies for both spleen weight and plasma [NO_x_] were similar across the year, in opposition from January to September and synchronized through December, suggesting a negative correlation followed by a positive correlation between spleen weight and plasma [NO_x_].

Three oscillations across the year were displayed by clotting time (Fig. 6, No *H*), capillary tHct (Fig. 13*G*, No *H*), and capillary *L*_p_ (Fig. 10*B*, No *H*), suggesting possible interconnections. The curves for *L*_p_ synchronized with tHct starting in March and with clotting time starting in May. Of note was the observation that *L*_p_ and tHct cycles correlated positively, and the relationship strengthened coincident with a unique feature of the *L*_p_ trace that showed oscillations increasing in amplitude as the year progressed. Positive coupling of capillary tHct with capillary *L*_p_ in situ represents a unique outcome from this study that has not been documented in the past.

A striking example of a sine wave with five oscillation cycles was the trace for capillary balance pressure (Fig. 12*A*, No *H*). The steady nature of the waveform across the entire year suggested internal controls with remarkable timing and precision. Although no statistical differences between months were observed, the time trace pattern was noteworthy for its rhythmic activity that most likely reflected the well-known “hunting” mechanism displayed by vascular smooth muscle, in this case, on the venous, downstream side of the capillary bed.

Liver weight (Fig. 2*C*, No *H*) approached a square wave curve. The trace was distinguished by an abrupt drop from winter to summer, sustained plateau in summer, and then a steep rise from fall into winter. We are not aware of any reports of a seasonal, >200% drop, and similar recovery in liver weight in *Rana pipiens*. This new information is fundamental to understanding annual processes of digestion, metabolism, and protein synthesis in this model.

A single saw tooth waveform was demonstrated by lymphocytes (Fig. 3*C*, No *H*) and capillary σ(π_c_–π_i_) (Fig. 12*B*, No *H*). The trough time points in March were similar between the two curves; however, each had a different rate of rise to peak. Lymphocytes displayed the steeper slope compared with the slower rise of capillary σ(π_c_–π_i_) with time. Speculation of a connection between these two variables was tempting. The initial increase in lymphocytes could signal an increase in σ(π_c_–π_i_) via changes in *J*_v_, *S*, and/or capillary pressure, which would increase resistance to water movement into tissues and, thus, preserve fluid in the vascular space during winter. In spring, lower lymphocytes could signal lower σ(π_c_–π_i_), which would reduce the resistance threshold required to move fluid out of the vascular space and, thus, restore tissue hydration. Experiments to test these ideas are required.

Flat line time series data for No *H* confirmed stability in the animal model. Steady heart rate (Fig. 5, No *H*) across the year was an index of the successful, consistent, low-stress housing environment that was provided for *Rana pipiens*. Capillary burst pressure (Fig. 12*D*, No *H*) also displayed little to no variation, indicating that capillary wall strength was maintained while dynamic changes in capillary *L*_p_ and σ(π_c_–π_i_) occurred. Heart rate and burst pressure plus the additional flat line curves listed in Tables 3 document important reference check points that are useful for assuring controlled studies of systemic and capillary physiology in *Rana pipiens*.

#### Rana pipiens with lung hematoloechus not attached (H Not Att).

None of the seasonal patterns discovered for No *H* were replicated in *H* Not Att. Instead, two variables associated with inflammation and four inflammation-responding variables were identified. First, plasma [NO_x_] (Fig. 4, *H* Not Att), which is inflammatory at high levels, approximated a double-peaked trace with a prominent elevation in spring (March, April, May). Second, eosinophils (Fig. 3*B*, H Not Att), known to be parasite responsive, approximated a saw tooth waveform that peaked from July into September. These results indicated that plasma [NO_x_] and eosinophils, together, effectively sustained inflammation from March through to September.

The first of the inflammation-responding variables was clotting time (Fig. 6, *H* Not Att), which displayed a sawtooth waveform that was inverse and shifted right relative to the time trace for eosinophils (eosinophil trough in January vs. clotting time peak in February; eosinophil peak in September vs. clotting time trough in November), suggesting possible stimulus-responses between these two variables. The second, third, and fourth inflammation-responding variables were capillary *J*_v_/*S* (Fig. 10*A*, *H* Not Att), capillary *L*_p_ (Fig. 10*B*, *H* Not Att), and capillary balance pressure minus σ(π_c_–π_i_) (Fig. 12*C*, *H* Not Att). Each displayed accentuated oscillations between March and September, indicating vigorous responses of capillaries during the same timeframe as the increases in plasma [NO_x_] and eosinophils. The accentuated intramonth variability of *J*_v_/*S*, *L*_p_, balance pressure minus σ(π_c_–π_i_) compared with No *H* and *H* Att was additional compelling evidence of disrupted/chaotic barrier control due to inflammation in *H* Not Att.

#### Rana pipiens with lung hematoloechus attached (H Att).

Heart rate, plasma [NO_x_], and *L*_p_ were the three variables with significant month-to-month differences in the annual time series data for *H* Att. From the heart rate trace (Fig. 5, *H* Att), it was clear that *H* Att were stressed at different time points during the year, a result that differed from the flat heart rate traces for No *H* and *H* Not Att. All animals in the study lived in the same controlled environment and received the same care, as such, an external source of stress was not apparent. Investigators were blind to presence or absence of *H* until necropsy. We conclude that the presence of attached *H* was likely an internal source of stress that was sufficient enough to influence heart rate. Seasonal changes in heart rate for *H* Att may be one of the best indicators of *H* activity/potency within *Rana pipiens*.

Plasma [NO_x_] (Fig. 4, *H* Att) also had a significant annual time series in *H* Att that was not present in No *H* and, when compared with *H* Not Att, was most instructive. The first prominent peak in spring that was the signature for plasma [NO_x_] in *H* Not Att was noticeably absent in *H* Att, and the second peak in August was muted. These traces suggested that *H*, when attached, introduced a nitric oxide-targeted inhibitor into the host. The high levels of plasma [NO_x_] in *H* Not Att during March, April, and May plus the absence of that peak during the same timeframe in *H* Att indicated the greatest proinflammatory (*H* Not Att) and anti-inflammatory (*H* Att) impact of *H*, an observation that could be useful for designing future experiments focused on nitric oxide, *H*, and *Rana pipiens*.

Surprisingly, *H* Att time series data revealed a seasonal change in capillary *L*_p_ (Fig. 10*B*, *H* Att). Once recognized, it was obvious that *H* attachment silenced an internal clock that was generating the *L*_p_ oscillations in No *H* as well as the accentuated excursions in *L*_p_ observed in *H* Not Att. The end result was a parasite-induced flat baseline for *L*_p_ with one internal clock still ticking, a seasonal change in *L*_p_ that was highest in January and lowest in November and that had gone undiscovered previously (28).

### Impact of Rana pipiens (Host) on Hematoloechus (Parasite)

Data also revealed an impact of host on parasite. Higher spleen weight in *H* Att suggested increased erythrocyte production; however, average [RBC] did not reflect an increase. This observation implied that, on average, increased RBC production kept pace with consumption in *H* Att resulting in [RBC] that remained at or near normal levels. However, [RBC] time trace data for all three parasite conditions (Fig. 7*B*) provided additional details and insight. The *H* Att [RBC] trace showed a deep trough in July that was absent in the other two groups indicating a point in the year when *H* Att were consuming host RBC vigorously and outpacing RBC production. Likewise, a deep trough in *H* Att skin lymph protein (Fig. 8*B*), a variable that also did not differ on average between groups, suggested active protein consumption in May. Together these data highlight specific feeding periods for *H*, when attached, and narrow the target for future studies focused on understanding feeding cycles of *H*. The data also suggest protein, in addition to RBC, as a potential source of nourishment for *H*.

### Secondary Analysis—Capillary *L*_p_ Outliers

During this 7-year study, we accumulated a dataset that was large enough to allow for separate analyses of variables from *Rana pipiens* that were associated with outlier values of *L*_p_. The added advantage was knowledge of *H* status, which allowed the data to be grouped accordingly.

Three distinct combinations or clusters of variables associated with the *L*_p_ outliers in each of the three parasite groups are listed in Table 1. In No *H*, a cluster of inflammatory variables was identified that reflected acute inflammation along with leakier and weaker capillaries. In contrast, none of the variables for No *H* were significantly different for *H* Not Att. Instead, decreased capillary density/capillary rarefaction was identified, indicating adaptation to chronic inflammation. The third scenario was in *H* Att, where lower spleen weight accompanied leaky capillaries.

The three unique outcomes for variables associated with *L*_p_ outliers support the assertion made by some past investigators that capillaries with high values of *L*_p_ are “inflamed”. However, it is important to note that the discovery of different variables for each group appears to reflect adaptation from acute to chronic inflammation related to specific stimuli associated with *H* status. We conclude that a high value of capillary *L*_p_ does not indicate similar etiology and is not necessarily a result of methodological anomalies. It is also worth noting that the third group (*H* Att) had the lowest “*n*” and the lowest median *L*_p_ of the three outlier groups further emphasizing the rarity of the data reported here and consistent with capillaries being less reactive to stimuli when *H* are attached. Finally, average total [WBC] were lower, not higher, for *L*_p_ outliers in No *H*, suggesting a minimum [WBC] that protects and maintains the integrity of the capillary barrier in *Rana pipiens*.

### Perspectives and Significance

For almost 100 yr, *Rana pipiens* has been valued for studies of capillary physiology (10, 29); yet, the broad set of variables presented here had not been characterized. Detailed measurements of variables at the system and individual capillary levels presented in the context of known *H* status are the first of their kind for *Rana pipiens*. We have discovered that *H* challenge *Rana pipiens* and, as a direct consequence, challenge the specialty of capillary physiology. Results intersect with the disciplines of medical and veterinary practices and helminthology, capillary physiology and pharmacology, and endothelial cell biology, biomedical engineering and mathematics. Examples are discussed below.

#### Medical and veterinary practices and helminthology.

The *H*-induced pro- and anti-inflammatory responses reported here focus the significance of this study on potential treatments for chronic inflammatory diseases such as cancer, asthma, obesity, Type II diabetes, Alzheimer’s disease, rheumatoid arthritis, and heart disease as well as parasitic diseases in animals and humans. In addition, increased capillary wall tensile strength with *H* Att indicates a mechanism that, if known and titrated properly, could be beneficial for preventing stroke/cerebral hemorrhage. *L*_p_ outlier data also demonstrated clearly that capillaries are leakier and weaker in the face of low [WBC], thus emphasizing a protective role of WBC, the mechanism/s of which would benefit patients with stroke and those with compromised immune systems.

The annual time series data provide more specific details of naturally occurring versus *H*-induced activity. Time lines of annual feeding cycles, prominent periods of inflammation, and high stress points due to *H* will allow for more fine-tuned investigations of *H* in the future. The data open possibilities for answering questions regarding how *H* regulates their feeding, how *H* stimulates proinflammatory and anti-inflammatory responses, how *H* stresses the heart only at certain times of the year, and how *H* strengthens capillary walls. The time series data also provide insight into the extended lengths of time necessary to induce effects in some variables. Excellent examples were capillary σ(π_c_–π_i_), which adjusted naturally over a period of 9 mo and *L*_p_ that required 11 mo to demonstrate adaptive change to *H* attachment. Each of these clues expand our collective knowledge about systems physiology and must be accounted for when translating this research into creative new ways to improve health and minimize the impact of disease.

*Schistosoma mansoni* is a water-borne trematode (blood fluke) with snail as its intermediate host and human is its definitive host. *S. mansoni* is the most prevalent parasite in humans causing the tropical disease, schistosomiasis. The fluke penetrates through the skin, lives in blood vessels, and comes into contact with endothelial cells during its migration through the body. In 1997, Coulson and Wilson (30) demonstrated increased inflammation in the presence of *S. mansoni* in the lung. In 1999, Trottein (31) studied the effect of *S. mansoni* on permeability of cultured endothelial cells originating from lung and brain. Their results indicated that *S. mansoni* secreted/excreted low molecular weight molecules that decreased monolayer permeability to inulin via cAMP/protein kinase A and phosphorylation of myosin light chain kinase producing an anti-inflammatory endothelium phenotype. These pro- and anti-inflammatory results from *S. mansoni* are consistent with our discoveries here with *H*. In *Rana pipiens*, we report: *1*) disappearance of oscillations in spleen weight, balance pressure, tHct, and *L*_p_ in *H* Not Att and *H* Att, *2*) disappearance of seasonal changes in σ(π_c_–π_i_) in *H* Not Att and *H* Att, *3*) highest *L*_p_ in *H* Not Att, *4*) highest [NO_x_] in *H* Not Att, *5*) lowest *L*_p_ in *H* Att, *6*) lowest [NO_x_] in *H* Att, and *7*) increased capillary wall tensile strength in *H* Att compared with No *H*. These results with the lung fluke (*H*) support the conclusion that *H* shift the in vivo endothelium from a pro- to anti-inflammation profile in *Rana pipiens* and are consistent with reports from blood flukes (*S. mansoni*). *S. mansoni* is well-studied, yet, how the host cells respond to the various proteins, lipids, glycans, and nucleic acids released by the blood fluke remains unknown (32). The results presented here introduce a new opportunity to investigate two different flukes that infest two different definitive hosts and produce similar outcomes. A comparative study may offer untapped avenues of discovery and insight into the mechanisms of parasite-host interactions.

#### Capillary physiology and pharmacology.

Our awareness expanded when we recognized that *H* and its attachment status were key to a more accurate understanding of capillary *L*_p_. Realizing that the *H* status was introducing “noise” into the overall *L*_p_ dataset was essential to acknowledging the fact that *H* was compromising our ability to glean important information about capillary barrier function in situ. By broadening our study, we discovered that *H* impacts, at a minimum, the immune and cardiovascular systems of *Rana pipiens*. We conclude that wild-caught *Rana pipiens* is a mixed model. We recommend that *Rana pipiens* be devoid of *H* in the future when used for scientific purposes and, in particular, for studies of capillary physiology. Adopting this recommendation, using the reference data presented here for No *H*, reporting *H* status (known or unknown) when referencing previous studies, and reporting *H* status in future studies will facilitate a new and higher “gold” standard for studies of single capillaries in situ and improve *Rana pipiens* as a long-standing, classic model.

Hydrostatic pressure, oncotic pressure, capillary permeability, and lymphatic clearance are fundamental to maintenance of tissue hydration, nutrition, and preventing edema formation. As such, the Starling forces (33) are integral to fluid balance and survival. By definition, balance pressure and σ(π_c_–π_i_) are obtained when flow through (balance pressure) and out of [σ(π_c_–π_i_)] the capillary are both zero. Calculating the difference between balance pressure and σ(π_c_–π_i_) pressure (Fig. 12*C*) from direct measurements provided new insight into the dynamic interplay between capillary pressure and barrier resistance to fluid filtration in situ (assuming constant precapillary resistance). The difference between the two revealed a more accurate prediction of whether fluid movement favored perfusion (delta pressure above zero) or filtration (delta pressure below zero). One practical example of how to apply this analysis is in the design of pharmacological agents. If a drug agent is engineered to manipulate only hydrostatic pressure without considering the impact on capillary barrier function, it is likely that side effects related to hydration, nutrition, and perhaps thermoregulation will ensue. This fundamental issue, when addressed, will minimize or negate some common symptoms associated with drug agents including thirst, weight loss or gain, and hyperthermia.

#### Endothelial cell biology, biomedical engineering, and mathematics.

Past investigators studying *L*_p_ of cultured bovine aortic endothelial cells (BAEC) have reported low and narrow baseline ranges for their systems. Some examples are the following: 1.65 to 4.94 × 10^−7^ (9); 3.1 to 4.4 × 10^−7^ (34); 2.87 to 2.97 × 10^−7^ (35); 2 to 5 × 10^−7^ (36); and 2.51 to 4.81 × 10^−7^ cm·s^−1^·cmH_2_O^−1^ (37).

Originally, Sill et al. (9) compared their data to values of *L*_p_ assessed in situ in frog mesenteric capillaries with median = 3.0 × 10^−7^, range = 0.87 to 25.74 × 10^−7^ cm·s^−1^·cmH_2_O^−1^ for *n* = 20 capillaries (38). In the present study, control *L*_p_ ranges were the following: 0.5 to 22.2 × 10^−7^ (No *H*); 0.7 to 43.9 × 10^−7^ (*H* Not Att); and 0.7 to 9.3 × 10^−7^ cm·s^−1^·cmH_2_O^−1^ (*H* Att) with low values that were not attained in BAEC and high values well above BAEC. In addition, Sill et al. (9) and Hillsley and Tarbell (36) excluded cultures with *L*_p_ >5.0 × 10^−7^ and > 6.0 × 10^−7^ cm·s^−1^·cmH_2_O^−1^, respectively, from their reports. No criteria were provided. The in situ frog mesenteric capillary data raise the question of how target baselines are selected for experiments on BAEC. Expanded rationale should be required in the future.

Mathematical models are used to test the theoretical impact of intercellular cleft dimensions, matrices, and meshes on *L*_p_ and solute permeability. Similar to the BAEC experiments, models have been grounded in in situ assessments of *L*_p_ in frog mesenteric capillaries. One group (8) tested their model using an average *L*_p_ value of 5.9 × 10^−7^ cm·s^−1^·cmH_2_O^−1^ from Clough and Michel (39) who also measured cleft dimensions on the same capillaries. Two years later, the same group (40) lowered the testing value for *L*_p_ to 2.0 × 10^−7^ cm·s^−1^·cmH_2_O^−1^ (41), a value that was three times smaller than their previous work. The reasoning for selecting the lower value for *L*_p_ was not provided.

Although using one number for *L*_p_ does not represent the dynamic nature of the capillary barrier, it is interesting to note that 5.9 × 10^−7^ cm·s^−1^·cmH_2_O^−1^ (39) is closer to the median for No *H* (7.0 × 10^−7^ cm·s^−1^·cmH_2_O^−1^) and 2.0 × 10^−7^ cm·s^−1^·cmH_2_O^−1^ (41) is more than three times lower than No *H* and more than two times lower than *H* Att (4.2 × 10^−7^ cm·s^−1^·cmH_2_O^−1^). We performed a comprehensive review of past capillary physiology studies based on whether results were conducted with or without the presence and effects of the *Hematoloechus* and found that none reported *H* status. We conclude that, in the past, low values of *L*_p_ likely were obtained from a mixture of *L*_p_ assessments performed in No *H*, *H* Not Att, and *H* Att plus *L*_p_ values > 10.0 × 10^−7^ cm·s^−1^·cmH_2_O^−1^ were excluded. The net result produced a left-skewed distribution of *L*_p_ and a false, low, target value of 2.0 × 10^−7^ cm·s^−1^·cmH_2_O^−1^ for baseline *L*_p_.

The present data provide insight into the implications of using a very low value of *L*_p_ for BAEC experiments and for testing mathematical models. Specifically, low *L*_p_ in *H* Att was associated with body weight loss in vivo (Fig. 1*A*). Although using *L*_p_ and cleft dimensions from the same capillaries is understandable from a modeling perspective, *L*_p_ and physiological parameters also must be considered if results are to be applied and translated into practice. This study provides corrected values for capillary *L*_p_ obtained from uninfected, uninfested *Rana pipiens* with known physiological parameters. We now recognize that a median *L*_p_ for No *H* of 7.0 × 10^−7^ cm·s^−1^·cmH_2_O^−1^ is more realistic for baseline capillary *L*_p_ as it reflects maintenance of body weight and a “healthy” endothelial phenotype. The corrected value is recommended for baseline *L*_p_ in BAEC and for tests of mathematical models in the future.

Because of our increasing awareness of the impact of *H* as the study progressed, some variables were added later than others. As such, datasets for clotting time, spleen weight, and liver weight were incomplete for *H* Not Att and *H* Att. However, because of the uniqueness of the data in *Rana pipiens* and for *H*, we have opted to include the data and allow readers to decide their value.

*Rana pipiens* used in this study were wild-caught and were infested with *H* naturally, not experimentally. The length of time that *H* were present in each frog was not known. *H* were identified by visual inspection, not genetically. Obvious patterns within data for each variable were not apparent, suggesting that genetically-identified subspecies of *H* and the different samples of *Rana pipiens* over 7 years did not impact the systemic and capillary variables studied here.

## DATA AVAILABILITY

Data will be made available upon reasonable request.

## GRANTS

Funding was provided by RO1 HL63125 National Institutes of Health (to D.A. Williams) and the Dean’s office, College of Nursing, Montana State University.

## DISCLOSURES

No conflicts of interest, financial or otherwise, are declared by the authors.

## AUTHOR CONTRIBUTIONS

D.A.W. conceived and designed research; D.A.W. and M.H.F. performed experiments; D.A.W. analyzed data; D.A.W. and M.H.F. interpreted results of experiments; D.A.W. prepared figures; D.A.W. drafted manuscript; D.A.W. edited and revised manuscript; D.A.W. approved final version of manuscript.
